# The Neuropsychological Assessment of Unilateral Spatial Neglect Through Computerized and Virtual Reality Tools: A Scoping Review

**DOI:** 10.1007/s11065-023-09586-3

**Published:** 2023-03-13

**Authors:** Stefano Terruzzi, Federica Albini, Gemma Massetti, Roberta Etzi, Alberto Gallace, Giuseppe Vallar

**Affiliations:** 1 Department of Psychology, University of Milano-Bicocca, Piazza dell’Ateneo Nuovo 1, Milan, 20126 Italy; 2 Mind and Behavior Technological Center, University of Milano-Bicocca, Milan, Italy; 3 Neurocognitive Rehabilitation Center (CeRiN), University of Trento, Rovereto, Italy; 4 School of Medicine and Surgery, University of Milano-Bicocca, Milan, Italy; 5 Neuropsychological Laboratory, Istituto di Ricovero e Cura a Carattere Scientifico Istituto Auxologico Italiano, Milan, Italy

**Keywords:** Information technology, Digital, Neuropsychological assessment, Unilateral spatial neglect

## Abstract

Unilateral Spatial Neglect is a disabling neuropsychological deficit. Patients with spatial neglect fail to detect and report events, and to perform actions in the side of space contralateral to a hemispheric cerebral lesion. Neglect is assessed by evaluating the patients’ abilities in daily life activities and by psychometric tests. Computer-based, portable and Virtual Reality technologies may provide more and precise data, and be more sensitive and informative, compared to current paper-and-pencil procedures. Studies since 2010, in which such technologies have been used, are reviewed. Forty-two articles meeting inclusion criteria are categorized according to their technological approaches (*computer-*, *graphics tablet or tablet-*, *virtual reality-based assessment*, and *other*). The results are promising. However, a definite golden standard, technologically based procedure cannot be still established. Developing technologically based tests is a laborious process, which requires technical and user experience improvements as well as normative data, to increase the evidence of efficacy for clinical evaluation of at least some of the tests considered in this review.

## Introduction

Unilateral spatial neglect (henceforth spatial neglect) is a disabling neurological disorder. Patients fail to report events (in different sensory modalities: visual, auditory, and somatosensory; when imaging a familiar visual scene from a given vantage point), which occur in the side of physical and imaginal space and the body contralateral to the side of the hemispheric lesion (contralesional). These patients also fail to perform actions in those sides of *extra-personal* and *personal* (i.e., the body) spaces (Chokron et al., 2008; Heilman et al., 2000; Vallar & Bolognini, 2014; Vallar & Calzolari, 2018; Vallar & Maravita, 2009). In most patients, neglect impairs the left side of the space, contralateral to a right-hemispheric lesion. Right spatial neglect, contralateral to a left-hemispheric lesion, is less frequent and severe (Bisiach et al., 1984; Bowen et al., 1999; De Renzi et al., 1970; Ogden, 1985). The reported incidence of spatial neglect after stroke varies across studies (ranging from about 10 to 90%). This wide range depends on a number of factors, including the operational definition of neglect, the patients’ selection criteria, and the assessment methods (Bowen et al., 2013). Spatial neglect is more frequent in the acute stage post-stroke (Bowen et al., 1999). After about three months, spatial neglect is still present in over 15% of right-brain-damaged patients (Ringman et al., 2004). In stroke patients the presence of spatial neglect predicts poor outcome in functional recovery (Di Monaco et al., 2011), entailing a longer time of hospitalization, greater functional dependency, increased risk of falls and a long-term disability in *Activities of Daily Living* (ADL) (Barer, 1990; Batchelor et al., 2012; Bernspång et al., 1987; Bowen et al., 2013; Czernuszenko & Czlonkowska, 2009; Nijboer et al., 2013). This results in an overall increase of costs for the health system (Barrett & Houston, 2019).

Spatial neglect is not due to lower-level sensorimotor deficits. Many patients show associated sensorimotor deficits, such as hemiplegia, hemianesthesia and hemianopia. However, a double dissociation (Teuber, 1955; Vallar, 2000) has been found between spatial neglect and sensorimotor deficits: patients may show sensorimotor deficits without neglect which, in turn, may be present in isolation (Bisiach et al., 1986). However, sensory and motor deficits may have higher-order components, brought about by spatial neglect itself (Vallar et al., 1991; Vallar, Sandroni, Vallar et al., 1991a, b). In line with these behavioral findings, the neural correlates of spatial neglect involve damage to higher-order associative areas, in perisylvian regions, including the inferior parietal lobule, the temporo-parietal junction, the posterior part of the superior temporal gyrus, the premotor cortex, white matter fiber tracts connecting these regions, the thalamus and the basal ganglia (Corbetta & Shulman, 2011; Karnath & Rorden, 2012; Ptak & Schnider, 2011; Vallar & Calzolari, 2018).

Spatial neglect may be conceived as a multifaceted syndrome with many clinical manifestations that frequently co-occur, but may manifest also in isolation (Halligan et al., 2003; Vallar, 1998). A primary distinction may be drawn between two sets of abnormalities: *defective* and *productive*. *Defective* signs, in which patients exhibit the absence of the appropriate behavior, requested by the experimental task or the ADLs, are the most investigated components of the syndrome. They include the impairment at attending to and exploring the left side of space, and of objects in it (Albert, 1973; Bisiach et al., 1981, 1983; Bisiach & Vallar, 2000; Gainotti et al., 1972; Rode et al., 1995; Schenkenberg et al., 1980; Stone et al., 1991; Vallar & Perani, 1986). Patients with spatial neglect may also show *productive* manifestations, that consist in gratuitous actions and delusional beliefs. These behaviors are inappropriate in the clinical setting, with respect to the requests by the examiner (as for *extra-personal* space: Kleinman et al., 2013; Ronchi et al., 2012, 2016; Rusconi et al., 2002; as for bodily space: Vallar & Bolognini, 2014; Vallar & Ronchi, 2009). Spatial neglect may occur in different dimensions or frames, with reference to the body and body parts (the mid-sagittal plane of the trunk, the head). In *egocentric* spatial neglect, the neglected side is coded with reference to the body’s trunk, the head, or the eyes. *Allocentric* spatial neglect refers instead to one side of an object, encoded with reference to its intrinsic coordinates (Howard, 1982; Klatzky, 1998, about these reference frames; see Vallar, 1998; Vallar & Bolognini, 2014 for these different manifestations of neglect). Furthermore, depending on the affected sector of space, spatial neglect may concern *personal* (the body), *near peri-personal* (the space surrounding the body, and within the reach of body effectors, primarily the hand, also the foot), and far *extra-personal* (not within the reach of such effectors) sectors of space (Vallar & Maravita, 2009). Finally, spatial neglect may also concern internally generated visual images (representational or imaginal spatial neglect, see Bisiach & Luzzatti, 1978; Bisiach et al., 1981; Meador et al., 1987; Grossi et al., 1989; Rode et al., 1995). All these manifestations of spatial neglect may occur in association, but dissociations among virtually all signs of the deficit have been observed (Bisiach et al., 1986; Guariglia et al., 1993).

### Quantitative and Standardized Assessment (“paper-and-pencil”) with Available Normative data

Up to the mid 1930’s (Weisenberg & McBride, 1935), the neuropsychological method coupled a qualitative, often insightful, analysis of the patient’s deficits with, whenever possible, a *post-mortem* examination of the patient’s brain, to determine the localization of the cerebral lesion, and to relate it with the observed symptoms and signs. Starting from the seminal observations by Broca about aphasia (Broca, 1861; Vallar & Caputi, 2021), the assessment of the patients’ behavioral deficits was qualitative, and consisted in the accurate observation of the behavioral deficits, which were typically apparent and, due to some peculiar features, had captured the attention of the examiner. The neuropsychological examination became then more and more based on standardized tests, which provide quantitative measures (i.e., scores) of the patients’ performances. Moreover, the performances of brain-damaged patients started to be compared, with the support of statistical procedures, with those of control participants, comparable to patients for demographic and socio-cultural variables, and differing from them only for the presence of the cerebral lesion and the putative deficit under investigation. The research work by the Italian neurologist Ennio De Renzi provides an example of this approach (Arrigoni & De Renzi, 1964; De Renzi, 1982). As to spatial neglect, the quantitative assessment of its clinical manifestations has traditionally relied on paper-and-pencil tests, which includes the tasks described in the following.

1) *Target cancellation*: to find out and mark targets printed on a sheet of paper. In the Line (Albert, 1973) and Circle (Bisiach et al., 1979; Vallar & Perani, 1986) tests only targets are displayed. In the Bells (Gauthier et al., 1989), Star (Wilson et al., 1987), and Letter (Diller & Weinberg, 1977) tests, targets are shown intermingled with distractors. Tests requiring target vs. non-target discrimination are more likely to detect spatial neglect than those in which only targets are present (Arduino et al., 2002; Vallar, 2000). Cancellation tasks, in which patients are required to discriminate between complete and incomplete targets (i.e., the incomplete ones with a left-sided or right-sided missing portion), allow to distinguish between *egocentric vs. allocentric*, stimulus-based, types of spatial neglect. In target cancellation tests, when left spatial neglect occurs in an *egocentric* reference frame (e.g., the mid-sagittal plane of the body), complete and incomplete targets, left-sided with reference to such a frame, are not crossed out. In *allocentric, object-based* left spatial neglect, targets with a left-sided missing part are not crossed out across the whole display, with reference to coordinates based on the target itself (Bickerton et al., 2011; Mancuso et al., 2015; Ota et al., 2001, 2003). Quantitative scores include the total number of omissions and the difference between the number of omissions in the two sides (left and right) of the display (a sheet of paper). Moreover, these tests may provide qualitative information also about other aspects of the patients’ performance, including: (i) the location of the first target crossed out; (ii) the directional pattern of exploration; (iii) the speed of execution, and (iv) the types of error. Errors include the *defective* omission of targets, that are not crossed out, and the *productive* manifestations: simple perseveration (repeated crossing out marks), and complex gratuitous productions (various types of drawings, e.g., a hen, the patients’ signature) (Gandola et al., 2013; Ronchi et al., 2009; Rusconi et al., 2002). Another measure of spatial neglect in cancellation tasks is the “center of cancellation”, consisting in the scaled mean position in the horizontal (left-right) dimension of cancelled targets (Rorden & Karnath, 2010; Toraldo et al., 2017).

2) *Bisection of a horizontal line*: to mark the location corresponding to the physical mid-point of a line, which is then divided it into two segments. In the standard version of line bisection patients set the mid-point using the not paretic arm and hand, ipsilateral to the side of the lesion (right-brain-damaged patients use their right hand, left-brain-damaged patients their left). Lines of different lengths may be presented, with their center aligned with the mid-sagittal plane of the participant’s body, or displaced leftward or rightward (Daini et al., 2002; Schenkenberg et al., 1980; Vallar et al., 2000). Typically, patients with spatial neglect set the mark shifted toward the end of the line ipsilateral to the side of the lesion (i.e., right-brain-damaged patients with left spatial neglect exhibit a rightward deviation from the geometrical mid-point of the line). Typically, the longer the line, the larger the bisection error (Schenkenberg et al., 1980; Vallar, 2000). The *Landmark* test (Milner et al., 1993) is a variant of manual line bisection, which does not require a motor response. In the Landmark test, in different trials participants decide which of the two halves of pre-bisected lines is longer or shorter, and communicate their response to the examiner, verbally or by pointing. In patients with left spatial neglect, deciding that the right half of the line is longer or shorter than the left half, independent of their actual lengths, and of the question posed by the examiner (longer or shorter?) indicates a response or output bias. By contrast, judging, erroneously with respect to the actual stimulus, that the left half of the line is shorter (and that the right half is longer) indicates a perceptual underestimation of the lateral extent of the left-sided portion of the pre-bisected line.

3) *Drawing*: to copy a meaningful or meaningless figure presented in front of the participant (Gainotti et al., 1972) or to draw a familiar figure from memory to a verbal command (Bisiach & Vallar, 2000; Rode et al., 2006). Patients’ performances are evaluated considering the extent of the omissions (complete or partial) of details in both the left- and the right-hand sides of the figure, or of each object, when the drawing includes multiple stimuli (Gainotti et al., 1972). Typically, drawings by patients with left spatial neglect are inaccurate and incomplete in their left-hand side. In copying figures including multiple objects, patients may omit either left-hand side objects, with reference to the mid-sagittal plane of their body showing, as discussed above, egocentric spatial neglect, or the left-hand side of each object, with reference to its coordinate frames, showing allocentric spatial neglect (Walker, 1995).

4) *Reading*: single words and non-words, sentences and passages of prose. Patients showing *neglect dyslexia* may commit errors of omission, substitution (Ellis et al., 1987; Kinsbourne & Warrington, 1962) and (less frequently) addition (Siéroff, 2017; Vallar et al., 2010) of letters and words (in reading sentences and passages of prose). Reading errors on words and orthographically legal non-words may be classified as neglect errors, using the “neglect point measure” (Ellis et al., 1987). Neglect errors in reading are defined as “errors in which target and error words are identical to the right of an identifiable neglect point in each word but share no letters in common to the left of the neglect point”. Left neglect dyslexia may be also detected using sentences (Antonucci et al., 1995). More ecologically-based reading material (i.e., passages of prose, as in articles or a menu) has been shown to be sensitive to detect the deficit (Galletta et al., 2014).

Patients with spatial neglect may also show a directional bias, or some other forms of neglect, in *Activities of Daily Living* (ADL). Prompted by several classical observations (see, for instance, Brain, 1941; Paterson & Zangwill, 1944), assessments based on the simulation of realistic conditions have been proposed to quantify the extent of spatial neglect in ADL. The *Behavioral Inattention Test* (Halligan et al., 1991; Wilson et al., 1987) is a comprehensive standardized battery that includes both conventional paper-and-pencil and behavioral subtests, in which patients are asked to perform tasks reproducing more practical and everyday life activities. Several batteries, including an ADL scale to evaluate extra-personal and personal spatial neglect have been proposed (McIntosh et al., 2000; Pizzamiglio et al., 1989; Zoccolotti et al., 1992; Zoccolotti & Judica, 1991). The *Catherine Bergego Scale* (CBS) assesses both the functional deficits in extra-personal and personal spaces (e.g., “forgets to eat food on the left side of his/her plate”; “forgets to clean the left side of his/her mouth after eating”), and the patients’ awareness of them, by comparing the observation by the examiner (the physician, the nurse, a relative) with the patients’ evaluation of their own behavior (Azouvi, 1996, 2002; Azouvi et al., 2003; Bergego et al., 1995). The CBS has been repeatedly reported to be a more sensitive tool to detect spatial neglect than the conventional paper-and-pencil tasks (Azouvi, 2002; Azouvi et al., 2003, 2006). Other ecologically valid and straightforward tasks used to assess spatial neglect in personal space require patients to reach contralateral body parts with their ipsilateral hand (Bisiach et al., 1986), or to explore their own body, using the ipsilateral unaffected arm and hand and, with eyes closed, to remove several Velcro stickers previously attached on their clothes, on both sides of the body (Cocchini & Beschin, 2020).

To summarize, a comprehensive clinical assessment of spatial neglect should include: (i) neuropsychological tests, evaluating both near extra-personal (target cancellation, line bisection, drawing by copy and from memory and reading tasks) and *personal* (body perception, body awareness, assessed by tests requiring patients to explore the two sides of the body, searching for body parts or objects on them) manifestations of the deficit (see Halligan et al., 2003; Menon & Korner-Bitensky, 2004; Vallar & Bolognini, 2014, for reviews); (ii) ADL scales or ecologic tasks, also assessing the patients’ awareness for the disorder (see for review Azouvi, 2017).

### Early Attempts to use Information Technologies (IT) for the Neuropsychological Assessment of Spatial Neglect

With increasing time from stroke onset, many patients recover, and may do well in neuropsychological tests, but still have difficulties in ADLs. A more complex control of spatial attention and awareness, and a major contribution from executive processes, may be required since, in everyday life, every activity is a multiple and not repetitive task, at variance from the previously discussed laboratory tests (Bonato et al., 2010, 2012; Della Sala et al., 2018).

Starting from the late 1990s, several computer- and touch-screen-based tasks have been devised and implemented, to provide a more sensitive and informative assessment, allowing to detect impairments in performance even in patients who do within the normal range in paper-and-pencil tests (Deouell et al., 2005; Erez et al., 2009; Rabuffetti et al., 2002; Schendel & Robertson, 2002).

Computerized tests have indeed some *pros*, typically recording more precisely information such as accuracy and response latencies throughout the execution of the task. To implement the architecture of the test devised by the experimenter, stimuli may be presented in different locations, simultaneously or as a sequence, with equal or varying time intervals between stimuli, across trials, sessions, or both, and may be repeated many times (Bonato et al., 2010; Buxbaum et al., 2012; Deouell et al., 2005). To manipulate the load posed to the participant’s resources, different levels of difficulty of the task can be easily implemented, varying for instance the duration and the number of the stimuli, and adding a concurrent task (Bonato et al., 2013; Bonato & Deouell, 2013). Additional behavioral measures may also be recorded, such as eye movements (Van der Stigchel & Nijboer, 2010), and data available from touch-screen recording (Rabuffetti et al., 2002), such as number and duration of touches, along with response latency measures recorded throughout the execution of the task (Kim et al., 2010). Furthermore, chances for a ceiling effect (namely, most patients obtain the maximum score in a test) are reduced. Quantitative, continuous measures, appropriate for statistical analyses in single patients, may be easily recorded (Robey et al., 1999), including sensitive individual monitoring of variations in performance levels through repeated assessments. Because of the possibility of easily producing unpredictable settings (e.g., presenting stimuli in random locations of the working space, with different shapes, and time duration), computerized tests can be made harder to learn, making more difficult the development of compensatory strategies on the patient’s part. Computerized tests are thus more suitable for test-retest designs, for instance readily allowing the assessment of the generalization effects of a given rehabilitation treatment (Geusgens et al., 2007). Finally, and importantly, computer-based tasks do not pose a general heavy burden on stroke patients in the post-acute and chronic phases. No adverse reactions, such as headache, fatigue, or boredom, have been reported. Conversely, digital tests have been evaluated as funnier, more precise and safer than the traditional paper-and-pencil ones (see, for instance, Pallavicini et al., 2015; Quinn et al., 2018; Smit et al., 2013; Ulm et al., 2013).

Also Augmented and Virtual Reality-CNADs should address all the above-mentioned issues. *Augmented Reality* (AR) can be defined as a set of techniques and tools, which add information to physical reality. AR has been used in many fields, including clinical psychology (Chicchi et al., 2015 for review), while its applications to neuropsychology are still limited. *Virtual Reality* (VR) is a computer-based multisensory, stimulating, and interactive environment, which occurs in real-time. Participants are engaged in activities that appear similar to real-world objects and events (Rizzo et al., 2004). VR can be subdivided in *non-immersive* (2-dimensional screen presentations, with interaction devices, such as a joystick), and *immersive* (requiring the integration of computers with further devices, such as Head-Mounted Displays, HDMs, VR controllers, or body-tracking sensors) applications. The *immersive* applications allow users to experience the virtual environment, and to interact with it, based on the movements of the head or body. For these reasons, starting from the 2000s, several VR paradigms have been implemented in both clinical and research settings (Neguț et al., 2016; Parsons, 2015; Rizzo et al., 2004). As compared to both paper-and-pencil and computer-based assessments, VR may be a useful tool to adequately evaluate ADLs in patients with spatial neglect, through scenarios that resemble everyday life situations (see for reviews Ogourtsova et al., 2017; Pedroli et al., 2015; Tsirlin et al., 2009). Several issues need however to be considered, although several neuropsychological VR paradigms have been developed in the last few years, facilitated by the decreasing costs of the hardware components and the increasing availability of open-access systems. Open issues include: (i) *VR-Induced adverse Symptoms and Effects – VRISE* (nausea, dizziness, disorientation, fatigue and instability). These symptoms and effects are mainly related to hardware and software inadequacies, not shared/overcome by more contemporary VR hardware and software (Kourtesis et al., 2019a, b). The virtual reality neuroscience questionnaire (VRNQ) is a tool which may facilitate the quantitative assessment of software attributes and intensity of VRISE (Kourtesis et al., 2019b). (ii) *Ergonomic features of VR tools*. Ergonomic and naturalistic interactions also minimize the risk of VRISE (Kourtesis et al., 2019a). Realistic interfaces, with direct hand interactions and wands with six degrees of freedom of movement, facilitate naturalistic and ergonomic interactions. Direct hand interactions are easier in terms of familiarization with their controls and efficiency and offer a more pleasant user experience; however, they appear less accurate than wands with six degrees of freedom of movement (Figueiredo et al., 2018; Sportillo et al., 2017). Stroke patients often use a wheelchair for locomotion, due to the presence of motor deficits, such as hemiparesis or hemiplegia. VR systems, that can be controlled with one hand (in patients with a hemispheric stroke, the non-paretic ipsilesional upper limb and hand), substantially improve the user experience of the tool itself (Fordell et al., 2011; Kim et al., 2011; Tsirlin et al., 2009). (iii) *Psychometric features of the developed systems*. Ecological validity is crucial for the assessment of spatial neglect because, with increasing time from stroke onset, patients may still show difficulties in ADLs, despite doing well in neuropsychological paper-and-pencil tests (Bonato et al., 2010, 2012; Della Sala et al., 2018). Immersive VR technology allows to collect data through the employment of dynamic stimuli and interactions with a high degree of control within an ecologically valid environment (Kourtesis et al., 2020; Rizzo et al., 2004). However, *immersion* depends on the *placement*, *plausibility*, and *embodiment illusions* (Maister et al., 2015; Slater, 2009, 2018). The placement illusion consists in the deception of users to be in a real and not in a virtual environment. The plausibility illusion is the deception of users that the environment reacts to their actions: they thus consider plausible that they are immersed in a real environment. The embodiment illusion refers to the deception that users own the body of the virtual avatar. (iv) *Competences.* Software developers and researchers are required to have adequate technological abilities, to opt for the appropriate hardware and software and to achieve their research, clinical aims, or both (Kourtesis et al., 2019a). Since a clinical staff typically has no programming skills, cooperation with software developers is needed, to develop and customize these systems (Fordell et al., 2011; Mainetti et al., 2013; Sedda et al., 2013; Sugarman et al., 2011). (v) *User experience.* This factor is affected by the used hardware, the quality of the sound and graphics, the level of immersion experienced by the user and the level of the enjoyment of the VR experience (Kourtesis et al., 2019b).

Notwithstanding the available evidence, the American Academy of Clinical Neuropsychology (AACN) and the National Academy of Neuropsychology (NAN), raise eight key issues regarding the development, dissemination and implementation of *Computerized Neuropsychological Assessment Devices* (CNADs), for both research and clinical aims: (i) safety and effectivity, (ii) identity of the end-user, (iii) technical features of hardware and software, (iv) privacy and data security, (v) psychometrics properties, (vi) examinee’s issues, (vii) use of reporting services, and (viii) reliability of responses and results. CNADs could be a standalone device or software, that can be run on a personal computer, a laptop, a tablet, or a smartphone (Bauer et al., 2012; see Kourtesis & MacPherson, 2021 for VR-related issues).

### The Review

Up to present, the manifestations of spatial neglect are mainly detected through neuropsychological paper-and-pencil tests, in both clinical and research settings. As noted above, in the subacute and chronic phases post stroke (Rehme et al., 2012), due to spontaneous recovery and cognitive rehabilitation, patients with spatial neglect may do well in neuropsychological tests, but still have difficulty in ADLs (Bonato et al., 2010, 2012; Della Sala et al., 2018). Information Technology (IT)-based devices (computer-, touch-screen- and VR-based) are useful tools for neuropsychological assessment, recording more information than paper-and-pencil tests and allowing to replicate and alter the surrounding world, to evaluate the patients’ behavior in contexts simulating real life (see for reviews Ogourtsova et al., 2017; Pedroli et al., 2015; Tsirlin et al., 2009). However, as previously mentioned, when developing, disseminating and implementing CNADs, for both research and clinical aims, several issues must be taken into account (Bauer et al., 2012; Kourtesis & MacPherson, 2021 for VR-specific issues).

The aim of this scoping review was to present an overview of studies from 2010 onwards, in which IT-based devices were used as a potential tool for the assessment of spatial neglect. Scoping reviews represent indeed a useful instrument to identify and map all available research on a specific topic, and to report information about how studies have been conducted (Munn et al., 2018). Only studies since 2010 onwards were included in this review for two reasons. Firstly, studies prior to 2010 have already been reviewed (Ogourtsova et al., 2017; Pedroli et al., 2015; Tsirlin et al., 2009); secondly, this review aimed at critically verifying whether the tools developed in the last decade successfully target the AACN & NAN Criteria (Bauer et al., 2012; see Kourtesis & MacPherson, 2021 for VR-related issues).

## Methods

The norms previously established by the PRISMA-P Group for scoping reviews (Tricco et al., 2018) were adopted. The protocol for this review was not previously registered.

### Search Strategy and Selection Criteria

The study search was initially kept broad to capture all relevant articles concerning the use of IT devices [computer, tablet and virtual (VR) or augmented (AR) reality], as possible tools for the clinical assessment of manifestations of the syndrome of spatial neglect. The following electronic databases were used for the identification of papers, up to July 2022: MEDLINE, ScienceDirect, Web of Science and Scopus. The search sting was: (“*technology*” OR “*computer*” OR “*tablet*” OR “*virtual reality*” OR “*augmented reality*”) AND [“*neglect*” OR (“*spatial neglect*” OR “*visual neglect*” OR “*visuospatial neglect*” OR “*hemispatial neglect*” OR “*unilateral spatial neglect*”)]. The reference lists of included articles and relevant reviews were checked to identify additional studies. The selection process was performed using *Rayyan – a web and mobile app for systematics reviews* (access from: https://www.rayyan.ai) (Ouzzani et al., 2016). Titles, abstracts, and full-text articles were screened independently by four out of the six authors (ST, FA, GM and RE), and evaluated for eligibility, based on the following inclusion and exclusion criteria:

- **Inclusion criteria**.
  - Related to, and making use of, any of the technologies employed in the clinical assessment of spatial neglect in brain-damaged patients (studies concerning nanotechnologies, technologies applied to eating behavior, nano-molecules, functional Magnetic Resonance Imaging - fMRI, Transcranial Magnetic Stimulation - TMS, transcranial Direct Current Stimulation - tDCS techniques and drug therapies were not included).
  - Focusing on adult brain-damaged patients with spatial neglect (i.e., at least one patient entered the study) and using the abovementioned technologies to assess neglect in patients with a clinical diagnostic aim.
  - Published since 2010.
  - Published in peer-reviewed journals.
  - Published in English.
- **Exclusion Criteria**.
  - Technical papers with no clinical application in patients with spatial neglect.
  - Literature reviews.
  - Unpublished data, conference proceedings or articles not reporting quantitative data.

These criteria are in line with those of other published reviews (Bucur & Papagno, 2021, 2022). A preliminary selection based on title, abstract and keywords was run, excluding those articles not matching selection criteria. A further selection was then made by inspecting full text manuscripts and applying inclusion and exclusion criteria. Unresolved papers were discussed by all authors, to reach a consensus. Duplicates were identified and removed through hand search.

In line with several review articles on neuropsychological topics (Bucur & Papagno, 2021, 2022; Ogourtsova et al., 2017; Zhang et al., 2017) we chose: (i) to include only papers published in English, (ii) not to include articles from gray literature (e.g., unpublished Ph.D. thesis) for two main reasons: (a) often, Ph.D. students embargo their dissertations, and (b) good level theses may be subsequently published in journals as research articles (see, for instance, Bucur & Papagno, 2019, 2022). With these inclusion and exclusion criteria, it is likely that only papers, which have passed the peer review process at an international level, are included in the present review.

## Results

**Fig. 1:**
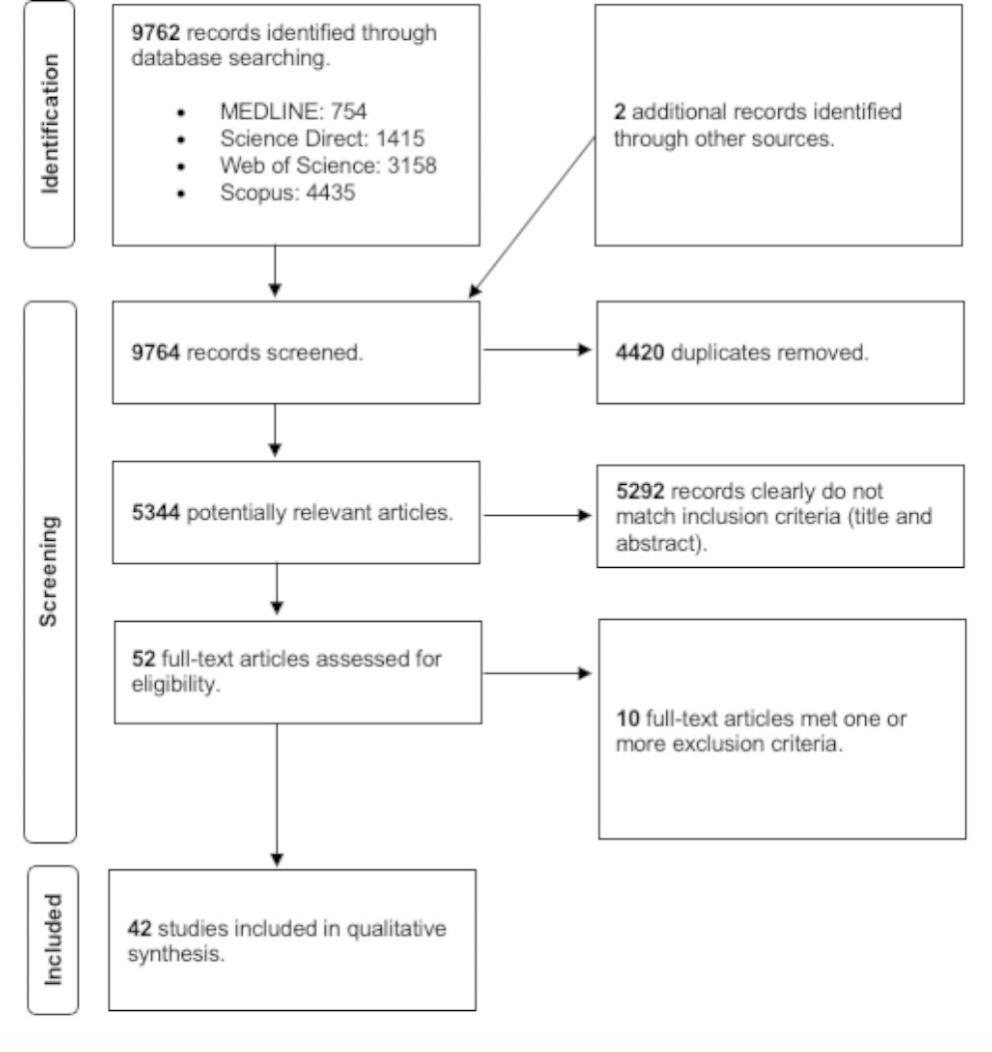
Flow diagram of study selection and inclusion criteria

The literature search retrieved 9764 articles. After duplicate removal, 5344 were screened for further evaluation because they were potentially relevant research articles, that explored the use of technologies (computer, tablet, VR or AR) as possible tools for the clinical assessment of spatial neglect. After Title and Abstract screening, 52 articles were assessed for eligibility, and, finally, 42 articles met all inclusion criteria and were further analyzed. Figure 1 shows the entire search and selection process.

Articles meeting the inclusion criteria were then categorized into four groups, depending on the digital instruments used to administering the digital tasks: *computer (pc and laptop)* (13), *graphics tablet and touch-screen tablet* (8), *virtual reality* (16), and *other* (5). The main features of the included studies are summarized in Table 1. In all studies, the presence/absence of spatial neglect was defined according to the neurological exam, a neuropsychological paper-and-pencil assessment, or both. Ten out of 12 studies describing *computer-based* tools included only patients suffering from a cerebrovascular attack (CVA). Two out these 12 studies (Bonato et al., 2013; Rabuffetti et al., 2012) included patients with different aetiologies (CVA, brain tumor, and traumatic brain injury). One study included also a control group of patients with Mild Cognitive Impairment (Blini et al., 2016). All 13 studies included right-brain-damaged patients; left-brain-damaged patients were included in six out of 13 studies (Blini et al., 2016; Kocanaogullari et al., 2020; Rabuffetti et al., 2012; Villarreal et al., 2020, 2021, 2022). All eight studies, describing *graphics tablet- and touch-screen tablet-based assessment* tools, included right-brain-damaged patients suffering from CVA; left-brain-damaged patients were included in two out of eight studies (Chung et al., 2016; Pierce et al., 2021). All 16 studies but one (Mesa-Gresa et al., 2011, which included also patients with a brain tumour) using *VR-based tools* included patients suffering from CVA. Left-brain-damaged patients were included in three out of the 16 studies (Aravind et al., 2015; Aravind & Lamontagne, 2014; Fordell et al., 2011). Immersive VR paradigms were used in all but two studies (Buxbaum et al., 2012; Grattan & Woodbury, 2017). Finally, among the *other* group, all the five studies included patients suffering from CVA and only two (Peru et al., 2017; Spreij et al., 2020) left-brain-damaged patients.

### Analysis of the Quality of the Studies

The quality of the included studies was analyzed using the *ad-hoc* quality criteria of Corti et al. (2021). Four of the authors (ST, FA, GM and RE) independently evaluated the quality of the studies; no discrepancies in ratings among the evaluators were found. The evaluation criteria, based on parameters considered as relevant for performing experimental and clinical neuropsychological studies, considered the presence (1 = yes, 0 = no) of:

1. *Sample size calculation*, based on power analysis, to avoid underpowered studies with unclear results, and the occurrence of publication bias. Recent literature points out the need to calculate the required sample size by using power analysis (Brysbaert, 2019; Kühberger et al., 2014). Specifically, for studies focused on tools for clinical neuropsychological assessment, power analysis can be performed by estimating the sample size needed to detect a significant difference in performance, with a specific power, with respect to a control group of participants not presenting impairments in the target construct, namely: healthy controls or patients without the impairment of interest (spatial neglect in the present review, see criterion below).
2. *Control group of healthy participants, of patients not presenting an impairment in the target construct, or of both*. The comparison of the patients’ performances with those of a control group of participants, matched for age, sex, and educational level, is a standard neuropsychological practice, to evaluate the performance of a patients’ sample with a specific diagnosis (in this review, spatial neglect), with respect to participants who do not show that specific impairment, namely: healthy control participants, patients without spatial neglect or both (Kan et al., 2019; Vallar, 2000). Statistical corrections, such as using co-variates in the analysis of variance (Capitani & Laiacona, 1997), can be performed to control for the possible effects of concomitant variables (age, sex, educational level, duration of disease in stroke patients, severity of the overall neurological impairment, etc.) on the patients’ performances in the tests assessing the target construct of interest (in this review, neglect).
3. *Sensitivity or specificity reporting. Sensitivity* is the ability of a test to correctly identify patients with a disease; *specificity* is the ability of a test to correctly identify people without the disease (Swift et al., 2020). Sensitivity and specificity are two psychometric properties that are used to assess target construct validity (i.e., the extent to which an instrument provides a measure of a theoretical construct).
4. *Measures of construct validity (the degree to which a test or instrument can measure a concept, trait, or other theoretical entity): convergent and divergent or discriminant validity reporting* (Strauss & Smith, 2009). Convergent or congruent validity is the extent to which responses to a test or to an instrument exhibit a strong relationship with responses to similar tests or instruments, namely the extent to which a measure correlates with other measures of the same or of a similar construct. *Divergent or discriminant validity* is the degree to which a test or measure diverges from (i.e., does not correlate with) another measure, whose underlying construct is conceptually unrelated to it.
5. *Ecological validity reporting.* Ecological validity indicates if the findings of a study can be generalized to realistic situations in everyday life. It is a subtype of *external validity*, that can be defined as the extent to which the measures of outcome correlate with, or predict, performance in ADLs (see also Holleman et al., 2020).
6. *User experience reporting*. This criterion was added to those proposed by Corti et al. (2021), to evaluate the presence of *negative* (i.e., heavy burden or adverse reactions, as headache, fatigue or boredom) or *positive* (i.e., feelings of fun and safety) reporting about the experience of using these digital tools, as compared to the traditional paper-and-pencil ones. This criterion was considered as met if user experience reporting was obtained from healthy participants, brain-damaged patients, clinicians, or from all of them.

The analysis of the quality of each study was then based on these six *ad-hoc* established criteria. With respect to sample size calculation based on power analysis, only two studies (Aravind & Lamontagne, 2014; Quinn et al., 2018) applied this procedure (4.76%). Twenty-six studies included a control group of healthy participants (61.90%), 19 had a control group of patients without spatial neglect (45.23%), and 12 included both (28.57%). As for psychometric properties, 23 studies reported sensitivity or specificity measures (45.23%), 20 convergent or discriminant validity (46.61%) and nine ecological validity (14.28%). Furthermore, among the studies reporting specificity or sensitivity, only two (Buxbaum et al., 2012; Chung et al., 2016) also reported the cut-off for impairment. This means that only for two out of 42 studies data to classify the patients’ performance as impaired or non-impaired (4.76%) were available. Finally, with respect to user experience reporting, only nine studies showed these data (21.42%). Table 2 shows the analysis of the quality of each study, based on the six criteria reported above.

**Table 1:** Characteristics of studies included. Summary of abbreviations and references:

COMPUTER							
Report	N: Sex (M/F); Age (M ± SD)	Aetiology. duration of disease	Conventional N Assessment	General Description	Technical aspects	User experience	Results
Chiba et al. (2010)	RBD (N+): 10 (6/4); 70 ± 8.51.	RBD: 10 CVA.	Drawing task (copy of a daisy)^12^; Line Bisection^20^; Line Cancellation test^25^.	1. *Verbal-Line Bisection (VLB) test*. 2. *VLB plus pointing test*. Horizontal black line presented on computer display. Character string (43 different Japanese language characters) subsequently superimposed on black line. Tasks’ sequence: (a) to identify the subjective midpoint of the line; (b) to read the character nearest to the line mid-point. In *VLB* the character read by participant is then pointed to by examiner using a cursor; participant required to confirm. In *VLB plus pointing*, participant points to the character with right index finger (proprioceptive feedback) and communicate if it is the one just read.	Stimuli presented on laptop computer, equipped with 39-cm liquid-crystal display. Windows operating system. Microsoft PowerPointⓇ software to show images. Computer placed on a table in front of participant, with centre aligned with participant’s body’s mid-sagittal plane (viewing distance about 45 cm).	-	In both the *VLB task* (8 out of 10 patients, 80%), and the *VLB task plus Pointing Task* (9 out of 10 patients, 90%), rightward deviation from the objective mid-point of stimuli.
Rabuffetti et al. (2012)	RBD (N+): 55 (32/23); 64.72 ± 14.5. RBD (N-): 66 (37/29); 56.1 ± 15.8. LBD (N-): 72 (41/31); 60.6 ± 14.2. HC: 119 (33/86); 58.92 ± 15.2.	RBD (N+): 48 CVA, 3 TBI, 3 BT, 1 Missing; 28 (1–3 m), 12 (4–12 m), 13 (> 12 m), 2 (missing). RBD (N-): 44 CVA, 11 TBI, 6 BT, 5 Missing; 24 (1–3 m), 12 (4–12 m), 22 (> 12 m), 8 (missing). LBD (N-): 56 CVA, 9 TBI, 3 BT, 4 Missing; 26 (1-3 m), 12 (4–12 m), 25 (> 12 m), 9 (missing).	BIT^3^’s Line Bisection; Letter Cancellation^18^; Line Cancellation^24^; Sentence Reading^32^; Wundt-Jastrow Illusion test^44^.	Visual stimuli (120: 40 targets, 80 distractors, letters, or shapes), presented on touch-screen monitor. Participants’ task: to touch with the index finger all targets. Sliding behaviour precluded by real-time testing software, allowing a touch-and-go behaviour. Maximum test duration: 10 min.	19” touch-screen monitor. Participants seated in front of screen with trunk’s mid-sagittal plane aligned with screen centre.	-	RBD N + patients slower and less efficient in search than HC, with more leftward and less downward direction of exploration. RBD N- patients: same pattern, halfway between RBD N + patients and HC.
Bonato et al. (2013)	RBD (N-, according to paper-and-pencil assessment): 10 (5/5); 67.	RBD: 9 CVA and 1 BT; 92 d.	BIT^3^ Line, Letter and Star Cancellation subtests.	Participants detect briefly presented lateralized targets in *Single Task* (ST) and *Dual Task* (DT) conditions. DT conditions: (i) *Visual Dual Task* (VDT, to report the identity of a centrally presented letter before reporting target position), (ii) *Auditory Dual Task* (ADT, counting by twos twice from a heard number, before reporting target position).	Participants seated at a 60-cm distance from a 15-in. computer monitor. Tasks programmed and administered using E-Prime (Bonato et al., 2010).	-	Computer-based tasks detect deficits in contralesional side of space in RBD patients, classified as N- by paper-and-pencil cancellation tests. Deficits more severe under DT (VDT and ADT) conditions: about 70% of contralesional targets not detected.
Ulm et al. (2013)	RBD (N+): 10 (6/4); 60 ± 8; HC: 10 (4/6); 68 ± 9.8.	RBD: 10 CVA. Of them, 5 (subacute group): 1.7 ± 0.8 m; 5 (chronic group): 20 ± 20.0.	CAV’s Computer-Based Extinction Test and examination of the visual field^9^; NET^28^’s Star Cancellation, Line Bisection, Figure Copying, and Clock Drawing subtests; DSS for visual and tactile extinction.	*Circle-Monitor (CM)* tests. 1. Star Cancellation test. 2. Line Bisection test. 3. Dice Task (DT). 4. Puzzle Test (PT). Star Cancellation and Line Bisection tests based on the same tasks in the NET^28^; DT, a simplified version of Baking Tray Task^2^; PT based on Hooper Visual Organization Test (HVOT)^17^. Participants completed all tasks using their right hand to reach the touch-screens, while sitting inside the CM. Total duration: about 10–15 min.	CM: eight touch-screens arranged in a circle and connected to computer outside the CM. Participants seated in the middle of the CM, at a distance adequate to reach monitors comfortably. Participants’ behaviour also supervised by a video camera.	CM’s tasks rated as more complex and fun, than paper-and-pencil versions, by N + patients and HC.	RDB patients’ performances at CM’s Star Cancellation and Line Bisection test comparable to those obtained in paper-and-pencil versions. More detailed analysis of time course of spatial exploration and additional scores (Crossing and Latency indexes) provided by CM. DT and PT subtests more sensitive to detect neglect than paper-and-pencil tests.
Jee et al. (2015)	RBD (N+): 11 (10/1): 63.54 ± 14.72. HC: -Group #1: 42 (21/22): 24.65 ± 2.87. -Group #2: 11 (5/5): 23.6 ± 2.18.	RBD (N+): 11 CVA, 6 m.	-	*Semi-computerized Line Bisection test (LBT)*. Twenty lines and dotted micro-patterns pre-printed with black ink on 21.5 × 28cm papers. Lines randomly selected and presented to participants with the instruction to cut each line in two equal halves, by placing a small pen notch through it, as closest to its centre as possible. Computed scores: (i) percent deviation, (ii) assessment duration (sec), (iii) neglected lines. Scoring: (i) LBT data sent to computer for computation and scores displayed on monitor, (ii) hand-drawn results given to two blinded raters, for independent scoring.	System installed on computer: position pattern recognizing the electronic pen, micro-pattern printed paper, installable LBT, assessment program. Written or drawn information detected and sent by the pen from a position-coded micro-pattern printed paper to computer, with LBT program installed.	-	Feasibility assessment of e-system first performed in HC, to evaluate intra- and inter-rater reliability. e-system then used in patients. Reliability of automatic scoring suggested by high inter-rater correlation between data scoring computed by automatic and manual procedures, the latter performed by two independent assessors.
Blini et al. (2016)	LBD (N-, according to neurological and neuropsychological exams): 10 (6/4); 53.2 ± 11.7. HC: 10; 65.8 ± 8.52. MCI: 8 (5/3); 69 ± 11.61.	LBD (N-): CVA, 456.1 ± 773.11 d.	BIT^3^’s conventional tests^2^. DSS paradigm ^11^ to assess visual half-field deficits and extinction.	Patients asked to report position of target(s), shown unilaterally or bilaterally on a computer monitor. ST and two DT conditions (visual vs. auditory). Target: a white disk, presented against a black background for 100 ms. Simultaneously with the lateralized target(s), visual shape shown at fixation point, or sound presented binaurally through earphones. ST condition: to report target(s) position. DT conditions: report both target position and visual central shape or presented sound.	Patients individually tested in quiet room, sitting at about 60-cm from 19-in computer monitor.	-	No spatial deficits in baseline neuropsychological assessment. Defective report of contralesional stimuli, both single and under conditions of DSS in the DT condition. No spatial bias in HC (performance at ceiling), and in MCI patients.
Mizuno et al. (2016)	RBD (N+): 2 (1/1); 62.5. RBD (N-): 1 M; 73. HC: 16 (11/5); 57.3 ± 10.7.	RBD (N+): 2 CVA, 7 w. RBD (N-): 1 CVA, 4 w.	BIT^3^’s conventional subtests.	Four visual exploration tests presented on a touch-screen monitor. 1. *Star Cancellation* test: cancelling targets by touching them. 2. *Circle* test: selecting complete circles, among incomplete ones. 3. *Visuomotor Search* test: to touch the screen where a Star target shows up in a random position. 4. *Visual Search* test: to push a button when a Star target shows up in a random position.	Participants seated at a desk in front of a 32-in. liquid crystal touchs-creen display, placed at a 45-cm distance from their eyes.	-	In Star and Circle tests, RBD N + patients slower than RBD N- patient and HC.
Machner et al. (2018)	RBD (Severe N+): 12 (9/3); 69 ± 3. RBD (Moderate N+): 12 (7/5); 68 ± 3. RBD (N-): 10 (3/7); 71 ± 3. HC: 11 (3/8); 69 ± 4.	RBD (Severe N+): 12 CVA, 5 ± 1 d. RBD (Moderate N+): 12 CVA, 4 ± 1 d. RBD (N-): 10 CVA, 4 ± 1 d.	Bells Cancellation test^5^; BIT^3^’s (German version) Line Bisection, Star Cancellation and Text Reading; CBS^7^; Figure Copying test^13^. CoC^8^ computed for Cancellation tests.	1. *Desk task.* Desk scene images (30 objects each, 100 trials) shown on 24-in. monitor. Participant’s task: to search for a paperclip (blue or red), to press a response button when the target was found, and to name its colour. Recorded data: detection rate, search duration and eye movements 2. *Posner Reaction Time (RT) task*. Computerized variant of Posner cueing paradigm (18.4-in. monitor). Data: detection rate and latencies.	1. *Desk task*. Participants seated at about 60-cm in front of a 24-in. widescreen thin-film transistor monitor: contact-free remote eye tracker running at 50 Hz. 2. *Posner RT task*. Notebook with a 18.4-in. thin-film transistor widescreen monitor.	-	Most RBD patients (also those classified as N- according to CBS and performing at ceiling at paper-and-pencil tests) show ipsilesional bias in *Desk Test*: fewer contralesional targets detected, and ipsilesional deviation of exploratory eye movements. High correlation of these rightward biased indexes with decreased detection rate and increased RTs for left-sided contralesional targets in *Posner RT task* and neglect-related functional impairment scores.
Rossa et al. (2019)	RBD (N+): 4 (1/3); 55.5 ± 17.2. HC: 8 (5/3); 49.37 ± 12.97.	Unavailable.	Bells Cancellation test^5^.	*READAPT* *Bells Cancellation test*: computerized version. Visual scenes made of 49 items (1 target, 48 distractors). In each trial participants communicate the target’s presence/absence, using the left/right mouse button. Three different versions: (i) *Standard* (one block, 80 trials). (ii) *Items* (five blocks, 14 trials each. Position of distractors on horizontal plane changed in every block). (iii) *Interference* (five blocks, 14 trials each. Percent degradation of the scene on the horizontal plane changed in every block). Eye movements recorded by eye tracker.	*REDAPT. W*eb application: a set of programs written in PHP and JavaScript, coupled with eye-tracking recorder. Participants seated in front of a computer in the straightest position possible, at a 60-cm distance from the screen. Eye tracker placed under the screen at a 30-45-cm distance from participants.	-	More target stimuli (total score and left-sided targets, not right-sided targets) omitted, and more time to complete the test by RBD left N + patients, compared to HC.
Kocanaogullari et al. (2020)	RBD (N+): 4 (3/1) 49 ± 10.96. LBD (N+): 1 (F) 57. RBD (N-): 1 (F) 68. LBD (N -): 4 (3/1) 68 ± 9.48. BILATERAL (N-): 1 (F) 69;	RBD (N+): 4 CVA, 42.33 ± 63.00 d. LBD (N+): 1 CVA, 7d. RBD (N-): 1 CVA, 17d. LBD (N -): 4 CVA, 10.2 ± 6.30 d. BILATERAL (N-): 1 CVA, 10 d.	BIT^3^	*Starry Night Test*. To press a key on a keyboard when detecting a visual target (a red dot) among distractors (smaller green dots), on a computer screen monitor. Response times recorded. EEG recording throughout the test.	Computer screen monitor with 17.23° by 9.74° viewing area. EEG recorded through 16 electrodes located at Fp1, Fp2, F3, F4, Fz, Fc1, Fc2, Cz, P1, P2, C1, C2, Cp3, Cp4, P5 and P6, according to 10–20 system with 256 Hz sampling frequency.	-	EEG data related to slower response to targets by patients. Three most informative EEG channels for detecting neglect: *Cz*, *P1*, and *F3* (broadly corresponding to regions in the anterior frontal cortex and the superior posterior parietal cortex). Response times not analyzed.
Villarreal et al. (2020)	RBD: 20 (9/11); 53 ± 8. LBD: 20 (15/5); 51 ± 9. HC: 20 (8/12); 46 ± 15.	RBD: CVA, 106 ± 45 d. LBD: CVA, 105 ± 45 d.	Bells Cancellation test^5^.	Two computerized DTs 1. *Detect task.* (i) To respond, by pressing a mouse button, to a red sphere flashing among other coloured ones in the peripheral visual field task. (ii) To respond when the digit “2” appears among different numbers presented in the centre of the screen. 2. *Crash task*. (i) To respond to a visual target (the collision of two moving grey spheres) in the peripheral visual field task (ii) To respond verbally when a number presented in the centre of the screen is twice as high as the immediately preceding one. In both peripheral and central vision, targets never presented simultaneously. Scores: correct responses, omission errors, RTs.	*Active Space*, based on near-field imaging technology. Several means of generating visual stimuli and measuring RTs. Main generator of visual stimuli: short-throw video projector producing a display 173-cm high and 277-cm wide (1.9 mm pixel size). Screen midpoint located 120-cm high from the floor.	-	More left-sided targets missed by RBD patients than by HC. No neglect in RBD, assessed by paper-and-pencil Bell Cancellation test. No difference in target omission between LBD and HC. No differences in RTs in both Detect and Crash tasks among RBD, LBD and HC.
Villarreal et al. (2021)	RBD: 20 (9/11); 53 ± 8. LBD: 20 (15/5); 51 ± 9. HC: 20 (8/12); 46 ± 15.	RBD: CVA, 106 ± 45 d. LBD: CVA, 105 ± 45 d.	Bells Cancellation test^5^.	Computer-based 1. *Lateralized target computer task*. Participants in front of a computer monitor at 60-cm distance. Three different stimuli presented concurrently: visual black dot target, visual central letter, and spoken number. Dot target shown in the left, or right hand-side of the screen, or bilaterally. Participants’ task: to report the position of the dot target(s), disregarding the central letter and the spoken number. 2. *Ball Rain Task*. To look at coloured spheres continuously appearing from the top of the screen in a fast downward motion. To respond, pressing the mouse button, only to red spheres.	15-inch computer monitor running E-Prime software. Video projector to generate visual stimuli, producing a 173 × 277-cm display on the wall (pixel size 1.9 × 1.9 mm). Task application implemented using LabVIEW™ systems engineering software.	-	1. *Lateralized target computer task*. With bilateral stimuli, more left-sided than right-sided target missed by RBD patients, than by LBD patients and HC. 2. *Ball Rain Task*. More targets contralateral to the side of the lesion missed by RBD and LBD patients than by HC. Higher RTs in patients. No spatial biases in Bells Cancellation test.
Villarreal et al. (2022)	RBD: 20 (9/11); 53 ± 8. LBD: 20 (15/5); 51 ± 9. HC: 20 (8/12); 46 ± 15.	RBD: CVA, 106 ± 45 d. LBD: CVA, 105 ± 45 d.	Bells Cancellation test^5^.	1. Large-screen version of the *Twinkle task*. 180 five-pointed star-shaped black outline figures arranged on a white background. 110 intact figures (distractors) and 70 figures with one point missing (target). Participants used a mouse with their dominant hand to move an on-screen cursor. Targets selected by pressing the left button of the mouse. 2. Finnish version of the *Visual* and *Auditory dual-tasks* developed by Bonato et al. (2010, 2013) (see lines above for task descriptions). Paper-and-pencil version of the *Twinkle Task* consisting of a screenshot of the large-screen version, with identical stimuli and proportions, presented on an A4 size paper.	*Active Space* was used to generate a large-screen version of the *Twinkle Task.* Main generator of visual stimuli: short-throw video projector producing a display 173-cm high and 277-cm wide. (see Villarreal et al. 2020). For technical aspects about the *Visual* and *Auditory dual-tasks* see General Description by Bonato et al. (2013)	-	Neither the computer nor the paper-and-pencil version of the Twinkle task was able to show spatial bias in RBD patients. In the Visual dual-task RBD patients missed significantly more left-sided targets than HC in both unilateral and bilateral trials. They also missed significantly more left-sided than right-sided targets only in the bilateral trials of the Auditory dual-task.
**GRAPHICS TABLET AND TOUCH-SCREEN TABLET**							
**Report**	**N: Sex (M/F); Age (M ± SD)**	**Aetiology; duration of disease**	**Conventional N Assessment**	**General Description**	**Technical aspects**	**User experience**	**Results**
Liang et al. (2010)	RBD (N+): 18. RBD (N-): 36. (Whole sample composed as follows: 30 M/24F; mean age: 75 ± 7.4.).	RBD (N+): 18 CVA. RBD (N-): 36 CVA.	BIT^3^.	Digitized tests 1. *Target Cancellation* (line, O/X). 2. *Figure Copying* (cross, cube, star, square). 3. *Figure Completion* (diamond, man, house). Participants, presented with half of a schematic figure split vertically, complete it. 4. *Drawing from Memory* (square, cube).	Test overlays placed and secured individually on Wacom UD1212 (A4) graphics tablet. Drawings executed using a cordless inking pen providing the same writing experience and user feedback as felt in a non-automated test procedure.	-	Performance of RBD patients, both N + and N-, at digital tests correlated with BIT scores. Overall efficiency of the testing process improved by the digital procedure: more indexes, compared to those of paper-and-pencil tests, extracted; scores automatically computed.
Smit et al. (2013)	RBD (N+): 31. RBD (N-): 2. (Whole sample: 19 M/14F; mean age: 60.36 ± 13.30).	RBD: 33 CVA, 63.73 ± 37.74 d.	-	Digitized tests performed on a tablet surface using a digital stylus. 1. *Object Cancellation.* 2. *Letter Cancellation* (scores: total number of omissions, total time for test completion, search time in ipsilesional and contralesional sides of display, horizontal and vertical CoC). 3. *Line Bisection* (distance of mid-point set by participant from physical mid-point; total time of execution).	22-inch interactive WACOM (PL2200) tablet screen. *DiagnoseIS* for programming. Tablet driven by laptop, to monitor stimuli and patients’ performance on the experimenter’s laptop.	-	Patients able to deal with the digital instrument, to comply with instructions, performing and completing the proposed tests within the allocated time frame indicating feasibility of computer-based assessment in CVA patients.
Pallavicini et al. (2015)	RBD (N+): 5/3. RBD (N-): 7/1. (Whole sample’s mean age: 66.1 ± 11.9).	RBD (N + and N-): 16 CVA, 20.31 ± 19.28.	BIT^3^’s Line and Star Cancellation subtests; Card-Dealing task from Semi-Structured Scales for the Functional Evaluation of Hemi-Inattention^29^.	*NeglectApp* 1. Two target cancellation tasks (with/without distractors corresponding, respectively, to the paper-and-pencil Line and Star Cancellation tests). 2. *Card-dealing task*, recreated in a Virtual Environment (VE), shown on the tablet.	*NeglectApp* tests carried out with a stylus for touchscreen on an iPad2 (screen size active area: 47.70 × 26.82-cm).	*System Usability Scale* (SUS)^38^. High usability of *NeglectApp*, as shown by the mean score of the whole sample.	Computer based *Line* and *Star Cancellation* tests as effective as paper-and-pencil versions in detecting left neglect. C*ard-Dealing* task more sensitive than functional scales to detect neglect.
Vaes et al. (2015)	RBD (N+): 20 (12/8); 61.65 ± 9.85. HC: 20 (9/11); 61.45 ± 9.37.	RBD (N+): 20 CVA.	-	D*igital Visuospatial Neglect Test Battery*, nine tasks. - Two *Cancellation* tests: digitized versions of the *Bells Cancellation* test^5^ and of the *Diamond Cancellation* test. - Two *Line Bisection* tests: a digitized version of Schenkenberg et al.’s (1980) *Line Bisection* test^14^ and a coloured *Rectangle Bisection* test. - *Visuospatial Search* task. - *Drawing by copy task*: alarm clock, complex butterfly. - V*isual DSS* test. - One spatial memory task: remembering 12 pictural stimuli placed to the left (six) and to the right of the display. - Visuospatial navigation test. After task execution, data processed online.	*Metrisquare DiagnoseIS* (MDIS) software package used to design stimulus pages, tasks, and the matching score system. A *DTU-2231 Wacom* (active area: 47.70 × 26.82-cm), with dual-screen technology. Participants used a pen at the display surface. Experimenters observed and reported the results at their computer screen.	-	Performance difference between left N + patients and HC, with patients performing worse for almost all indexes extracted by the digital apparatus, with a rightward bias: CoC in target Cancellation tasks; Centre of Drawing CoD) in the Drawing test; Centre of Navigation (CoN) in the visuo-spatial navigation test.
Chung et al. (2016)	RBD (N+): 20 (10/10); 69.2 ± 10.2. RBD (N-): 10 (2/8); 65.5 ± 13.0. LBD (N-): 10 (5/5); 61.1 ± 12.0. HC: 10 (5/5); 46.0 ± 15.2.	RBD (N+): 20 CVA, 8.0 ± 6.3 d. RBD (N-): 10 CVA, 4.0 ± 1.8 d. LBD (N-): 10 CVA, 6.8 ± 6.6 d.	Line Bisection test^23^; Star Cancellation test^37^. Total Neglect Score calculated from scores of the two tests.	*Computer Table Setting Test (CTST*). A virtual table with 12 dishes shown in the middle of the screen. Participants set the table by dragging individual plates, located below the table, to the top of the table. Three parameters extracted: horizontal deviation from the midline, selection tendency for dishes on the right, elapsed time to set the table.	*CTST* developed to run on iPad.	-	Overall sensitivity of the test higher than that of paper-and-pencil *(*Line Bisection, Star Cancellation) tests. RBD N + and N- patients discriminated by scores in the horizontal deviation parameter.
Willer et al. (2016)	Experiment #1: Patients: 54 (34/20); 63.8. HC: 48 (22/26); 67.5. 30 patients and 11 HC from the sample above were then re-tested. Experiment #2 Patients: 16 (11/5); 67.3.	Experiment #1: Patients: 54 CVA, 17 d. Experiment #2: Patients: 16 CVA, 48.4 d.	BIT^3^’s Line, Star and Letter Cancellation subtests; DKEFS^10^’s Color-Word Interference test; WAIS-IV^41^’s Digit Span and Coding subtests; Western Aphasia Battery (WAB)^43^ (part II b and IV a); WMS-IV^42^’s Symbol Span and Designs subtests.	*Cognitive Assessment at Bedside for iPad (CABPad).* Digitized versions of: Rating of Anosognosia, Motor Speed for Hands, Speech Comprehension, Picture Naming, Verbal Fluency (phonemic and semantic), Timed Neglect Test, Baking Tray Test, Attention Span (symbols), Working Memory (symbols), Arrow Stroop, Episodic Memory (pattern locations), Symbol Digit Coding, GDS^16^ short form. Phase 1: patients and HC tested twice with a one-month interval Phase 2: patients tested with CABPad and conventional tests for neglect.	*CABPad*, developed *by* Palle M. Pedersen. Made available in Apple’s App Store.	-	Medium-to-high correlations between almost all *CABPad*’s subtests and paper-and-pencil tests. Performance of left N + patients worse than that of HC in *Timed Neglect*, but not in *Baking Tray* subtest.
Quinn et al. (2018)	Sample: 48 patients (28/20); 63. (3 N + according to gold-standard assessment; no more data available for the side of lesion site).	Sample: 48 CVA, 38 d.	*Object Bisection* test. Six horizontal lines, drawn offset from the center. Goldman or Octopus perimetry’s Visual Field assessment. *Line Cancellation* test^24^.	*StrokeVision ap*p. Assessment of sensory (visual acuity, visual field), and spatial and exploratory (line bisection, target cancellation) processes.	App used with a Google Nexus 10 tablet device.	0–10 scale (0 = completely unacceptable, 10 = perfect acceptability), administered to both clinicians and patients.	Feasibility and acceptability for stroke patients: app-based assessment completed by patients; modality of testing found acceptable.
Pierce et al. (2021)	RDB (N+): 12 (10/2) 62 ± 11.33. RDB (N-): 17 (12/5) 60.06 ± 8.8;1. LBD (N-): 10 (5/5) 59.7 ± 12.26. HC: 14 (6/8) 66.14 ± 7.87.	RDB (N+): 12 CVA, 9.47 ± 5.79 m. RBD (N-): 17 CVA, 11.03 ± 8.69 m. LBD (N-): 10 CVA, 4.65 ± 1.94 m.	Bells Cancellation test^5^; Line Bisection test^22^; Apples test^1^. Performance scored as the difference in target omissions on the left vs. right side of the sheet. CoC^8^ measure also scored.	*Manual exploration task.* Digitized task, running on a tablet, assessing manual spatial exploration. Participants, blindfolded, tap on the screen using their index finger until the target (a rectangular area 1/8 by 1/5 of the screen size that shows up in a random location on each trial) is found. When the target is tapped, a chime sound is played, and the target moves to a new location. Duration of each trial: 40 s.	Touch screen tablet computer (Microsoft® Surface Pro 4; 26 × 17-cm screen area; 2736 × 1824 pixel resolution) running a custom MATLAB® (Mathworks, Natick, MA) script.	-	Four out of 12 RBD patients, classified as N + by paper-and-pencil tests, show a significant rightward shift of the average horizontal position in the *Manual exploration task*, compared to both N- patients and HC. In six patients the rightward shift differs only from the shift of HC. No correlation between shift in the *Manual Exploration Task* and errors in the paper-and-pencil Bells and Apples Cancellation tasks and shift in Line Bisection.
**VIRTUAL REALITY-BASED ASSESSMENT**							
**Report**	**N: Sex (M/F); Age (M ± SD)**	**Aetiology;** **duration of disease**	**Conventional N Assessment**	**General Description**	**Technical aspects**	**User experience**	**Results**
Kim et al. (2010)	RBD (N+): 16 (10/6); 52.9 ± 16.8. RBD (N-): 16 (11/5); 60.1 ± 12.1.	RBD (N+): 16 CVA. RBD (N-): 16 CVA.	Letter Cancellation test^19^; Line Bisection test^22^.	*Three-Dimensional Virtual Street Crossing Program Contents*. Participants, immersed in the VE, required firstly to place in front of them an avatar presented in the centre of the screen; then, asked to make the avatar safely cross the street by stopping a virtual car that could approach the midline at four different velocities; participants could receive visual or auditory cue. 16 missions performed.	A personal computer system with a 3D graphics acceleration card and speakers used to create the VE. Participants immersed in the VE by means of a head-mounted display (HMD). Participants’ head movements measured using a head tracking system with 3 deg. of freedom in the VE.	-	Significant rightward deviation from the midline in locating the avatar by RBD left N + patients, compared to RBD N-patients. Percent deviation in Line Bisection test correlates with deviation from midline in the VR-based task. RTs of N + patients higher when the virtual car is approaching from the left hand-side of the screen; failure rate (i.e., virtual car not detected by the time the avatar’s position is reached, despite cues), higher than those of RBD N- patients.
Tanaka (2010)	RBD (N+): 9 68.7 ± 7.61.	RBD (N+): 9 CVA, 4–27 w.	BIT^4^’s (Japanese version) Line and Star Cancellation subtests; modified version of Halligan et al.’s (1991) checklist of everyday neglect behaviours.	Paper-and-pencil Line and Star Cancellation tests replicated in a VE. Paper tests photographed by a digital camera. Images altered, by increasing (zoom-in condition) or reducing (zoom-out), their size and displayed in the HMD.	Digital camera, HMD and digital video camera, recording participants’ head movement as qualitative motion analysis.	-	VR cancellation tests: lower scores in the left-sided than in the right-sided halves of the VR working sheet area in all RBD left N + patients. Only in three out of 9 patients, scores lower than the cut-off in paper-and-pencil Line and Star Cancellation tests.
Fordell et al. (2011)	N+: RBD: 8; LBD: 1. 6/3; 73.3 ± 12. N-: RDB: 11; LDB: 11. 16/6; 74.4 ± 10.8.	N+: 9 CVA. N-: 22 CVA. (Duration of disease range for the whole sample: 1–20 w).	Baking Tray Task^2^; BIT^3^; Line Bisection^21^; Star Cancellation test^36^; Visual Extinction test^40^.	*VR-DiSTRO* battery: four subtests, developed into an interactive and immersive 3D experience, evaluating neglect by: (i) Baking Tray Task (VR-BTT); (ii) Star Cancellation Task (VR-SCT); (iii) Line Bisection (VR-LB); (iv) Visual Extinction (VR-EXT). Total time for the assessment: about 15 min.	Desktop computer with stereo-capable graphics card, stereo headphones, robotic pen, separate numeric keyboard, 19” CRT monitor, shutter glasses for stereoscopic vision. Glasses, monitor, and software create a virtual 3D world. Using the pen, the patient’s hand perceives “*virtual*” touch sensation and realistic force feedback.	Overall, satisfaction in the experience, and full comprehension of instructions, reported by patients. No reported adverse effects.	In all tasks, rightward spatial exploration bias in RBD N + patients. For *VR-DiSTRO*, very high values of sensitivity and specificity for detection of neglect. For performances in Baking Tray and Visual Extinction tests the highest correlation with BIT total score, and high sensitivity and specificity. Lower sensitivity for Star Cancellation and Line Bisection tests of the *VR-DiSTRO*.
Mesa-Gresa et al. (2011)	Patients: 25 (14/11); 51.2 ± 12.62. (According to BIT scores, N+: 5; N-: 20).	Patients: 22 CVA and 3 BT (L or R), 505.42 ± 335.11 d.	BIT^3^; Colour Trail Making Test (CTT); Conners’ Continuous Performance Test (CTT-II).	V*R Street Crossing Test (VRSCT)*. Task: to cross a virtual two-way road to arrive at a destination point and then to come back to the starting point. When an accident occurs, patients receive emotional audio-visual feedback and new instructions. Session completed when patients perform two complete routes with four or less accidents. Measures: number of times participants look leftwards and rightwards; total time needed to complete the task; total number of accidents; completion/non-completion of the task.	Conventional panoramic 47” LCD monitor and 5.1 surround sound system. Interaction with and navigation in VE, by conventional joystick. Optical system to track patient’s head movements.	-	In RBD left N + patients higher number of accidents than in RBD N- patients, with no left vs. right differences. No correlation between *VRSCT* and N + patients’ BIT scores.
Peskine et al. (2011)	RBD patients (7 N+): 9 (5/4); 50 ± 15. HC: 9 (5/4); 50.6 ± 16.1.	RBD patients: 9 CVA; 16.11 ± 30.16 m.	Bells Cancellation test^6^; CBS^7^.	Participants immersed in a VR town, in which they can move forward via a mouse click. Patients, sitting in a swivel chair, have to turn on their vertical axis, to change their point of view and moving direction. Task: locating the primary target (swings in a park) and counting bus stops (6 on one side and 7 on the other side of the street), along the way.	Participants equipped with HMD. coupled with electromagnetic sensor system.	-	More targets and more left-sided bus stops omitted by RBD patients than by HC. Convergent results between VR-based tasks and paper-and-pencil tests only in 3 out of 12 patients.
Sugarman et al. (2011)	RBD (N+): 1 (F); 66.	RBD (N+): CVA; 15 m.	Paper-and-pencil Cancellations tasks (no details available about specific tests).	*VR React task* of the *SeeMe System*: virtual balls appear randomly on both sides of the screen. Task: to touch the virtual ball within a set amount of time. Number of missed balls, and movement time to reach the target, recorded by the system.	*SeeMe*: projected video capture, VR system with algorithms for movement and position recognition and analysis. Single screen-mounted camera, and vision-based tracking system, to capture and convert participant’s movements for processing.	Short Feedback Questionnaire (SFQ)^34^. Patients gave a full score of 5 out of 5 for the items of “enjoy”, “feeling of control” and “success in the virtual tasks”.	Performance of RBD patient in paper-and-pencil tests within normal range. In *VR React task* half of left-sided and none of right-sided targets missed. Patient’s average movement time to targets presented in left side of screen higher than that to right-sided targets.
Buxbaum et al. (2012)	RBD patients: 70 (39/31); 59.5 ± 10.6. HC: 10 (5/5); 62.2 ± 15.1.	RBD patients: 70 CVA, 29.2 ± 23.7 m.	Modified version of Fluff test^14^; timed version of Bells Cancellation test^5^; BIT^3^’s Letter Cancellation and Line Bisection subtests; Buxbaum et al.’s (2004) Laser Line-Bisection task; modified version of Buxbaum et al.’s (2008) Moss Real World Navigation (RWN) test.	*Virtual Reality Lateralized Attention Test (VRLAT)* (Buxbaum et al., 2008; Dawson et al., 2008): participants travel along a virtual, nonbranching path, either propelling themselves using a computer joystick (*participant condition*), or passively viewing the environment while an examiner navigates the path at a constant rate (*examiner condition*). Participants asked to identify virtual objects (coloured trees and statues of animals) on either side of the path, and to avoid colliding with them. Array conditions: (i) *simple* (contains only target objects); (ii) *complex* (contains targets and distractors); (iii) *enhanced* (as the complex condition, with the addition of auditory and small visual moving distractors).	*VRLAT* run on a PC. Participants seated 34 in. away from a 15.5 by 27.5-in. flat-screen video display, interfaced with the *VRLAT* via a Logitech® Attack 3 joystick. Software on the Unreal Engine 2 programmed by Digital Media Works.	HC and 64 patients completed all six conditions of the *VRLAT*. Six patients unable to complete the *participant-driven conditions*, due to difficulty in operating the joystick.	Based on performances on paper-and-pencil tests, 32 (50%) out of 64 patients classified as left N+. This percentage increases to 36 (56.3%) using the VRLAT. In the *Moss Real World Navigation* test eight RBD patients make left-sided collisions. All eight patients classified by *VRLAT* as N+; only five out of eight patients classified as N + by paper-and-pencil tests.
Dvorkin et al. (2012)	Experiment #1: RBD (N+): 8 (4/4); 59 ± 12.20. RBD (N-): 9 (8/1); 59.6 ± 7.61. HC: 9 (5/4); 55.7 ± 8. Experiment #2: RBD (N+): 3 (2/1); 50.33 ± 11.23. HC: 2.	Experiment #1: RBD (N+): 8 CVA, 18.62 ± 20.87 m. RBD (N-): 9 CVA, 64.11 ± 41.40 m. Experiment #2: RBD (N+): 3 CVA; 20.33 ± 15.04.	BIT^3^’s conventional subtests.	*Virtual Environment for Spatial Neglect Assessment (VESNA)*. Experiment #1: on each trial, participants instructed to press a response button, when a target is detected. 105 static targets distributed on a Cartesian grid, from right to left of body mid-line, above, below, and at eye level, and in peri-personal and far extra-personal space. Experiment #2: 35 targets distributed on a polar grid, a fan-like pattern of targets centred between the participant’s eyes, to assess whether spatial deficits are influenced by the rectangular arrangement of targets.	Images displayed by cinema-quality digital projector. Over 5-foot-wide 1280 × 1024 pixel image resulting in a 110° wide viewing angle. Head motion tracked by sensors attached to stereo shutter glasses worn by participants, sitting in a dark room on a chair placed in front of system and holding a response button in their right arm. Head position kept firm by chin rest.	-	All RBD patients classified as N + by VR paradigm. Only 50% of patients classified as N + by BIT. In the *VESNA*, HC detect all targets. In left N + patients higher RTs and lower detection rate for left- than for right-sided targets, and for near than for far targets.
Aravind & Lamontagne (2014)	RBD (N+): 10 (3/7); 59.9 ± 8.55. LBD (N+): 2 (1/1); 64.5 ± 10.60.	RBD and LBD (N+): 12 CVA; 13.56 ± 24,28 m.	Bells Cancellation test^5^; Line bisection test^22^.	*Virtual Environment*. A room with dimensions matching those of a physical room. A blue circular target presented on the wall at the far end of the virtual room; three red cylinders (obstacles) placed in front of a possible point of collision. 1. *Locomotor task*. Patients to walk towards the blue target, avoiding collisions with approaching moving obstacles, coming from the centre, left- or right-hand sides of VE. Control trials devoid of any moving obstacle. 2. *Perceptuo-motor task*. Patients seated, responding using a joystick. Task: to press the joystick button when an obstacle is detected, and not to respond in the absence of obstacles.	*Locomotor* and *Perceptuo-motor tasks* performed while patients view the Virtual Environment by nVisorSX60 HMD. Positions of three reflective markers placed on HMD, tracked by 12-camera Vicon-512™motion capture system and fed to CAREN 3™ VR software, to provide the real-time update of the patients’ perceived position and orientation in the Virtual Environment.	-	1. *Locomotor task*. Five out of 12 patients collide with contralesional, eight with middle and none with ipsilesional obstacles. Patients’ performances unrelated to clinical measures of neglect (Bells Cancellation, Line Bisection tests). Fewer collisions in slower walkers. 2. *Perceptuo-motor task*. Longer latencies in patients to detect contralesional vs. ipsilesional obstacles.
Aravind et al. (2015)	RBD (N+): 10 (3/7); 59.9 ± 8.55. LBD (N+): 2 (1/1); 64.5 ± 10.60.	RBD and LBD (N+): 12 CVA; 13.56 ± 24,28 m.	Bells Cancellation test^5^; Line Bisection test^22^; Motor Free Visual Perceptual Test (MVPT)^26^.	*Virtual Environment* as in Aravind & Lamontagne (2014). Tasks: 1. O*bstacle Detection Task*. Patients, by clicking a joystick button, respond on perceiving the onset of obstacle motion). 2. L*ocomotor Obstacle Avoidance Task.* 3. J*oystick-Driven Obstacle Avoidance Task*. Patients proceed towards a target and avoid oncoming obstacles.	See above Aravind & Lamontagne (2014).	-	1. O*bstacle Detection Task*. Contralesional and head-on obstacles detected at closer proximities than ipsilesional obstacles. 2. L*ocomotor Obstacle Avoidance Task.* Collisions only when approaching contralesional and head-on obstacles. 3. J*oystick-Driven Obstacle Avoidance Task*. For contralesional obstacles approaching, patients’ avoidance strategies start at shorter distances from the obstacle; minimum distances from contralesional obstacles are smaller than for ipsilesional obstacles.
Grattan & Woodbury (2017)	RBD patients: 12 (6/6); 67.7 ± 8.8.	RBD patients: 12 CVA; 617.5 d ± 852.7.	BIT^3^’s Star Cancellation, Line Bisection subtests and all the BIT^3^’s behavioural subtest (BIT-bs); CBS^7^; Naturalistic Action Test (NAT)^27^.	Buxbaum et al.’s (2012) *VRALT’s enhanced* array (distractor objects, auditory distractors, and moving distractors) in *examiner condition* (the examiner navigates the pathway using the joystick), because some participants are unable to operate the joystick.	*VRLAT* run on a PC laptop using a Logitech Extreme 3D Pro joystick.	-	Neglect detected in six out of 12 RBD patients by both the conventional and the behavioural subtests of the BIT, in all 12 patients by the *VRALT*. *VRALT* scores correlate with CBS and NAT scores.
Aravind & Lamontagne (2018)	RBD (N+): 13; 59.8 ± 7.7. RBD (N-): 13; 60.8 ± 6.5. HC: 5 (3/5); age range: 50–74.	RBD (N+): 10.5 ± 4.6 m. RBD (N-):11.8 ± 5.1 m.	Apples test^1^; Bells cancellation test^5^; letter cancellation test^18^; line bisection test^22^; MVPT^26^.	1. *Locomotor Obstacle Avoidance Task*. 2. *Perceptual Task*. See above Aravind & Lamontagne (2014).	See above Aravind & Lamontagne (2014).	-	Collisions with left and head-on obstacles and longer latencies for detecting left vs. right obstacles by left RBD N + patients. More walking time spent by N + patients with head rotated rightwards, towards the ipsilesional side, as compared with RBD N- patients. In RBD left N + patients, rightward deviations while walking in all obstacle conditions. N + patients fail to perform early or large enough adjustments to their heading and walking speed in response to contralesional obstacles, with increased risk of collisions.
Ogourtsova et al. (2018a, b)	RBD (N+): 12 (10/2); 60 ± 8.8. RBD (N-): 15 (13/2); 58.5 ± 13.2. HC: 9 (4/5); 56.3 ± 11.2.	RBD (N+): 12 CVA, 1,7 ± 1,1 y. RBD (N-): 15 CVA, 2.0 ± 2.1 y.	Apples test^1^; Line Bisection^22^; Star Cancellation^36^.	*Ecological VR-based Evaluation of Neglect Symptoms (EVENS).* Immersive and 3D navigation test. Virtual presentation of a symmetrical and richly textured room, displaying a grocery shopping aisle with three shelves located in front and 3 m away from participants. Target and distractors conditions. 1. *Simple (*target stand-alone in five possible locations). 2. *Complex* (target intermingled with distractors. Tasks. 1. *Detection* (participants press the joystick button as soon as they detect the target). 2. *Navigation* (*p*articipants navigate using a joystick, controlling speed to reach the target with the most direct possible trajectory. Maximal Medio-Lateral Deviation (mMLD) from the best navigation trajectory computed.	VE created in the Unity® Game Engine. Viewing media: HMD, blocking all peripheral vision, with only VE visible to participants. Responses provided with the right hand, using a stationary joystick.	Focus groups and questionnaire, directly involving expert clinicians, shows their interest and availability to use an immersive 3D tool.	D*etection task*. In RBD N + patients, compared with RBD N- patients and HC, higher detection times for left-sided and middle targets.
Ogourtsova et al. (2018a, b)	RBD (N+): 15 (12/3); 60.2 ± 8.8. RBD (N-): 15 (13/2); 58.5 ± 13.2. HC: 15 (7/8); 61.0 ± 11.8	RBD (N+): 15 CVA, 1.6 ± 1.0 y. RBD (N-): 15 CVA, 2.0 ± 2.1 y.	Apples Test ^1^; Line Bisection test (LBT)^22^; Star Cancellation test (SCT)^36^. LBT and SCT performed in the near (40 cm) and far (320 cm) space through a screen; participants’ responses provided by a laser pointer.	*VR-based Navigation* and *Detection tasks* performed with participants seated and immersed in a 3D VE, representing a symmetrical and richly textured room, including a visual display of walls and ceiling. Target: a red ball, presented 7 m away from the starting navigation position in five possible locations, in different conditions: (1) remains visible during movement, (2) disappears after an initial auditory cue is presented, (3) moves rightwards or leftwards.	VE created in Softimage XSI®. During trials, scene controlled by real-time CAREN-3™ software. Viewing media: a helmet-mounted display. Responses provided by joystick.	-	In all *VR-based Navigation* conditions, more errors, overshooting left-sided targets, by RBD left N + patients vs. HC and RBD N- patients. In the *Detection task*, longer detection times for all target location in RBD N + patients, vs. HC and RBD N- patients. In RBD N + patients, longer detection times for the L target compared to the R target, as well as for L and R targets compared to the middle ones.
Knobel et al. (2020)	RBD: 15 (7/8); 67.1 ± 10.05. Of them, N+: 10; N-: 5. HC: 35 (28/7); 69.0 ± 7.47.	RBD: 15 CVA; 76.5 ± 36.0 d.	Sensitive Neglect Test (SNT)^30^.	*Virtual Cancellation Task (VCT)*. A blue background and 120 objects (20 white-coloured spheres and 100 cubes), which changed their colour to red when touched with the controller. Participants find targets and reach and touch them using the hand-held controller and avoid distracters.	VR setup: an HMD and a hand-held controller, connected to a gaming laptop.	Cybersickness rated very low (SSQ)^35^. Very high ratings of acceptance and usability (SUS)^38^.	RBD N + patients perform worse than RBD N- patients and HC in both the VCT (higher total time and lower percent detected targets) and in the SNT. In RBD N + patients, CoC, displaced more leftward when measured by paper-and-pencil than by VCT. VCT detects neglect as reliably as paper-and-pencil cancellation tasks, then when measured by the VR cancellation task, the VR cancellation task could detect N as reliably as the used paper-pencil cancellation task.
Kim et al. (2021)	RBD (N+): 19 (14/5) 54.32 ± 7.40. RBD (N-): 22 (17/4) 49.23 ± 9.99. HC (10/12): 45.41 ± 17.82.	RBD (N+): 19 CVA, 11.79 ± 12.37 m. RBD (N-): 22 CVA, 10.57 ± 10.96 m.	Line Bisection test (LBT)^22^; Star Cancellation test (SCT)^36^; CBS^7^.	*FORP Task*. VR-based technique to assess *Field of Perception* (*FOP*) and *Field of Regard* (*FOR*) in N + patients. For *FOP* and *FOR* tasks, participants instructed to click the left or the right button of computer mouse, to a blue (left-sided click response) or red (right-sided response) target, presented in VE. *FOP Task*: targets detected in the absence of head movement. *FOR Task*. head movements for target detection allowed.	*FOPR Tasks*: stereo HMD system and 3D development platform controlled by desktop workstation running Windows 7, equipped with high-end graphics card. Screen resolution: 1280 × 800. Built-in 3-deg-of- freedom sensor to track head movements.	-	Differences detected by *FOP* and *FOR Tasks* in percent successful responses and response temporal indexes. RBD N + patients perform worse than both RBD N- patients and HC. Good agreement between *FORP Tasks* and paper-and-pencil Line Bisection and Star Cancellation tests and CBS.
**OTHERS**							
**Report**	**N: Sex (M/F); Age (M ± SD)**	**Aetiology;** **duration of disease**	**Conventional N Assessment**	**General Description**	**Technical aspects**	**User experience**	**Results**
Sotokawa et al. (2015)	RBD (N+): 3 (2/1).	RBD (N+): 3 CVA.	Japanese version of BIT^4^.	*Driving-Simulator Lane Tracking task.* To keep the lane continuously while driving on a road with random curves. The ratio of straying from the course measured and calculated as error ratio. Duration of Driving-Simulator test: about 10 min.	Mitsubishi Inc.’s® Precision Driving Simulator (DS-20).	-	Ratio of steering from the route biased leftwards. Interpretation: recovery from left neglect, with leftward overcompensation, possibly supported by *pseudoneglect* (i.e., the physiological minor leftward bias in line bisection, shown by healthy participants) (Jewell & McCourt, 2000). No neglect at the BIT.
Peru et al. (2017)	RBD (N+): 6(2/4). LBD (N+): 1 F. (patients’ age range: 62–81). HC: 7.	RBD (N+): 6 CVA. LBD (N+): 1 CVA.	Bells Cancellation test^5^; BIT^3^’s Copying, Drawing and Line Bisection tests; Line Cancellation test^24^; Sentence Reading test^31^.	A uniform distribution of stain images, differing in number (24 or 48) and features (coffee or water stains), and dust grains displayed on the top of an interactive table. Participants detect and cancel out stains using a round sponge. Stimuli reproduce stimulus size and spatial arrangement of papery Albert’s (1973) test. Each stain cancellation carried out under two conditions: (1) similar to papery Albert’s (1973) test (the spot, when “touched” by the sponge is marked by a red line); (2) more “ecologic” (the spot disappears, as the sponge touches it.	Interactive table developed by University of Florence’s Media Integration and Communication Centre (MICC). Table device equipped with a 52 multi touch-screen monitor similar in shape to a dinner table with a wood surface-like.	**-**	Flawless performance of 4 out of 7 RBD patients on the papery version of Albert’s (1973) test. Left-sided omission errors in the interactive table version by all seven patients. Patients’ performance overall better in the erasing than in the marking condition. HC: errorless performance.
Montedoro et al. (2019)	Patients: 35 (24/11) (RBD: 23; LBD: 12); of them, 25 diagnosed with N+. (patients’ mean age: 62.63 ± 11.89). HC: 56, paired by mean age.	All the patients: 35 CVA, 11.17 ± 12.30 m.	Apples test^1^; Bells Cancellation test^5^; Computerized Neglect subtest of the Test Battery for Attentional Performance (TAP)^39^.	*MonAmour Task*. Target search task. Participants find a grey colored musical conductor (target), randomly intermingled among 119 black colored musical instruments of four different types (distractors). Targets complete or incomplete (in their left- or right-hand side), measuring then egocentric vs. allocentric neglect. Participants, when a complete target is detected, make a push response using the end-effector of the *REAplan*®*robot* towards the conductor’s baton; when an incomplete target is detected, they make a pull response, towards the conductor’s musical stand.	The REAplan®robot used for the development of the MonAmour test. REAplan®: a planar end-effector robot with position and force sensors allowing for precise action measures in the horizontal plane (dimensions 93 × 52-cm).	**-**	For egocentric neglect, patients’ performance in computer-based task highly related to performance in paper-and-pencil tests, for left-right asymmetries in omissions and response latencies. Excellent sensitivity of *MonAmour* test, compared to both *VRLAT* (Buxbaum et al., 2006; Dawson et al., 2008) and *VR-DiSTRO* (Fordell et al., 2011). For allocentric neglect, strong correlation between *MonAmour* test and the Apples test; better sensitivity and specificity of *MonAmour* test than the Apples test.
Cerrato et al. (2020)	RBD: 9 (6/3). HC: 115 (54/61); 27.2 ± 8.6.	RBD: 9 CVA, 48.55 ± 55.33 m.	Bells Cancellation, Copy of a Landscape Drawing, Overlapping Figure and Line Bisection tasks from the GEREN neglect battery^15^.	*E-TAN technology Platform*. Enhanced version of Baking Tray Task^2^ (*E-BTT*). Tangible physical interfaces: 4-cm in diameter, to be placed on the platform. The polygon area defined by the spatial collocation of the disks on the platform indexes surface exploration. Timing and temporal sequence of collocations recorded.	30 fps camera used to detect disks’ position over the platform. Marker applied above each disk, recognized by ArUco Markers system.	Some patients spontaneously describe the task as agreeable and quick to perform, and as a welcome change after so many boring paper-and-pencil tests.	Great sensitivity of *E-BTT* to mild patial deficits, due to novel recorded measures, based on the use of the convex hull described by objects on the board. This measure provides an estimate of the portion of space processed by participants and may effectively discriminate N + vs. N- patients. No reported comparisons between performances in paper-and-pencil test *vs. E-TAN.*
Spreij et al. (2020)	RBD (N+): 33 (22/11); 58.83 ± 9.18. LBD (N+): 7 (6/1); 54.75 ± 11.48. RBD (recovered-N): 7 (4/3); 54.47 ± 14.69. RBD and LBD (N-): 53 (40/13); 58.86 ± 12.14. HC: 21 (11/10); 58.77 ± 9.86.	RBD (N+): CVA, 60.36 ± 31.83 d. LBD (N+): CVA, 43.0 ± 26.54 d. RBD (recovered-N): 7 CVA, 50.0 ± 37.76 d. RBD and LBD (N-): 53 CVA, 41.89 ± 39.77 d.	Shape Cancellation task (SC)^33^: 54 targets shapes presented among 75 distractors on a computer screen monitor. Participants instructed to find all targets and to click on them: a circle appears on the screen around the location of the mouse click. CBS^7^.	*Simulated driving task* (van Kessel et al. 2010, 2013). A driving scene projected on a large screen. No car interior shown; a steering wheel fixed on a table in front of participants, instructed to use it to maintain the starting position at the centre of the right lane. Simulated driving speed approximately 50 km/h at a set pace. Position in the lane, manipulated by simulated “side wind” from either side, needs to be adjusted continuously by participants. The projection of the driving scene vibrates as a warning signal, when participants drive off the road into the left or right verge. Task duration: two min.	Driving scene projected on a large screen (2.13 x 3.18-m), with participants seated in front of the screen, placed about 90-cm from their eyes.	-	Left N + RBD patients (assessment performed by the SC and the CBS) deviate leftward, contralesionally, towards the neglected side of the street, more than RBD N- patients. Interpretation: result possibly brought about by asymmetries in the layout of the simulated driving task.

*Participants*: Healthy Controls (HC); LBD/RBD (Left/Right-Brain-Damaged patients); MCI (patients with Mild Cognitive Impairment); (N+/-) Patients with/without spatial neglect. VE/VR (Virtual Environment/Reality).

*Etiology and duration of disease*: Cerebrovascular attack (CVA); brain tumor (BT); traumatic brain injury (TBI). d/w/m: days/weeks/months, years.

*Conventional Tasks and Batteries*: ^1^Apples test (Bickerton et al., 2011); ^2^Baking Tray Task (Tham, 1996); ^3^Behavioral Inattention Test – BIT (Wilson et al., 1987); ^4^Behavioral Inattention Test – BIT (Isiai, 1999); ^5^Bells cancellation test (Gauthier et al., 1989); ^6^Bells cancellation test (Isiai, 1999); ^7^Catherine Bergego Scale – CBS (Azouvi et al., 2003); ^8^Center of cancellation – CoC (Rorden & Karnath, 2010); ^9^Computer-Based Assessment of Visual Function – CAV (Niedeggen & Jörgens, 2005); ^10^Delis-Kaplan executive function system – DKEFS (Delis et al., 2012); ^11^Double Simultaneous Stimulation test – DSS (Bisiach & Faglioni, 1974; Làdavas, 1990); ^12^Drawing task (Ishiai et al., 1989); ^13^Figure copying task (Ogden, 1985); ^14^Fluff test (Cocchini et al., 2001); ^15^GEREN neglect battery (Azouvi et al., 2006); ^16^Geriatric Depression Scale – GDS (Yesavage & Sheikh, 1986); ^17^Hooper Visual Organization Test – HVOT (Hooper, 1983); ^18^Letter cancellation test (Diller & Weinberg, 1977); ^19^Letter cancellation test (Weintraub & Mesulam, 1988); ^20^Line bisection (Levine et al., 1986); ^21^Line bisection (Wilson et al., 1987); ^22^Line Bisection Test – LBT (Schenkenberg et al., 1980); ^23^Line Bisection Test – LBT (Lee et al., 2004); ^24^Line cancellation (Albert, 1973); ^25^Line cancellation (Levine et al., 1986); ^26^Motor Free Visual Perceptual Test – MVPT (Colarusso & Hammill, 1972); ^27^Naturalistic Action Test – NAT (Schwartz et al., 2002); ^28^Neglect-Test – NET (Fels & Geissner, 1997); ^29^Semi-Structured Scale For The Evaluation of Personal and Extrapersonal Neglect (Zoccolotti et al., 1992); ^30^Sensitive Neglect Test – SNT (Reinhart et al., 2016); ^31^Sentence reading (Barbieri & De Renzi, 1989); ^32^Sentence Reading (Zoccolotti et al., 1989); ^33^Shape Cancellation task – SC (Van der Stoep et al., 2013); ^34^Short Feedback Questionnaire (Rand et al., 2008); ^35^Simulator Sickness Questionnaire – SSQ (Kennedy et al., 1993); ^36^Star cancellation test (Wilson et al., 1987); ^37^Star cancellation test (Halligan et al., 1989); ^38^System Usability Scale - SUS (Brooke, 1996); ^39^test battery for attentional performance – TAP (Zimmermann & Fimm, 2002); ^40^Visual Extinction Test (Geeraerts et al., 2005); ^41^Wechsler Adult Intelligence Scale – WAIS-IV (Wechsler, 2012); ^42^Wechsler Memory Scale – WMS-IV (Wechsler, 2009); ^43^Western Aphasia Battery – WAB (Kertesz, 1982); ^44^Wundt-Jastrow illusion test (Massironi et al., 1988).

**Table 2:** Evaluation of the quality of the studies

COMPUTER							
Report	Sample size calculation based on power analysis	Presence of healthy control group	Presence of N- control group	Sensitivity/Specificity reporting	Convergent Validity/Discriminant Validity reporting	Ecological Validity reporting	User experience report
Chiba et al. (2010)	0	0	0	0	0	0	0
Rabuffetti et al. (2012)	0	1	1	1	0	0	0
Bonato et al. (2013)	0	0	0	0	0	0	0
Ulm et al. (2013)	0	1	0	1	1	0	1
Jee et al. (2015)	0	1	0	1	0	0	0
Blini et al. (2016)	0	1	0	0	0	0	0
Mizuno et al. (2016)	0	1	1	0	0	0	0
Machner et al. (2018)	0	1	1	1	1	1	0
Rossa et al. (2019)	0	1	0	0	0	0	0
Kocanaogullari et al. (2020)	0	0	1	1	0	0	0
Villarreal et al. (2020)	0	1	0	1	0	1	0
Villarreal et al. (2021)	0	1	0	1	0	0	0
Villarreal et al. (2022)	0	1	0	1	0	0	0
**Total studies: N = 13**	**0/13 (0%)**	**10/13 (76.92%)**	**4/13 (30.76%)**	**8/13 (61.53%)**	**2/13 (15.38%)**	**2/13 (15.38%)**	**1/13 (7.69%)**
**GRAPHICS TABLET AND TOUCH-SCREEN TABLET**							
**Report**	**Sample size calculation based on power analysis**	**Presence of healthy control group**	**Presence of N- control group**	**Sensitivity/Specificity reporting**	**Convergent Validity/Discriminant Validity reporting**	**Ecological Validity reporting**	**User experience report**
Liang et al. (2010)	0	0	1	1	1	0	0
Smit et al. (2013)	0	0	1	0	0	0	0
Pallavicini et al. (2015)	0	0	1	1	0	1	1
Vaes et al. (2015)	0	1	0	0	0	0	0
Chung et al. (2016)	0	1	1	1	1	0	0
Willer et al. (2016)	0	1	0	0	1	0	0
Quinn et al. (2018)	1	0	0	1	0	0	1
Pierce et al. (2021)	0	1	1	1	1	0	0
**Total studies: N = 8**	**1/8 (12.5%)**	**4/8 (50%)**	**5/8 (62.5%)**	**5/8 (62.5%)**	**4/8 (50%)**	**1/8 (12.5%)**	**2/8 (25%)**
**VIRTUAL REALITY**							
**Report**	**Sample size calculation based on power analysis**	**Presence of healthy control group**	**Presence of N- control group**	**Sensitivity/Specificity reporting**	**Convergent Validity/Discriminant Validity reporting**	**Ecological Validity reporting**	**User experience report**
Kim et al. (2010)	0	0	1	0	1	0	0
Tanaka (2010)	0	0	0	0	1	0	0
Fordell et al. (2011)	0	0	1	1	1	0	1
Mesa-Gresa et al. (2011)	0	0	1	0	1	0	0
Peskine et al. (2011)	0	1	0	0	0	0	0
Sugarman et al. (2011)	0	0	0	0	0	0	1
Buxbaum et al. (2012)	0	1	0	1	1	1	1
Dvorkin et al. (2012)	0	1	1	1	0	0	0
Aravind & Lamontagne (2014)	1	0	0	0	1	0	0
Aravind et al. (2015)	0	0	0	0	1	0	0
Grattan & Woodbury (2017)	0	0	0	1	0	0	0
Aravind & Lamontagne (2018)	0	1	1	0	1	0	0
Ogourtsova et al. (2018a, b)	0	1	1	1	1	0	1
Ogourtsova et al. (2018a, b)	0	1	1	0	1	0	0
Knobel et al. (2020)	0	1	1	1	1	0	1
Kim et al. (2021)	0	1	1	1	1	1	0
**Total studies: N = 16**	**1/16 (6.25%)**	**8/16 (50%)**	**9/16 (56.25%)**	**7/16 (43.75%)**	**12/16 (75%)**	**2/16 (12.5%)**	**5/16 (31.25%)**
**OTHERS**							
**Report**	**Sample size calculation based on power analysis**	**Presence of healthy control group**	**Presence of N- control group**	**Sensitivity/Specificity reporting**	**Convergent Validity/Discriminant Validity reporting**	**Ecological Validity reporting**	**User experience report**
Sotokawa et al. (2015)	0	0	0	0	0	0	0
Peru et al. (2017)	0	1	0	1	0	0	0
Montedoro et al. (2019)	0	1	0	1	1	0	0
Cerrato et al. (2020)	0	1	0	0	0	0	1
Spreij et al. (2020)	0	1	1	1	1	1	0
**Total studies: N = 5**	**0/5 (0%)**	**4/5 (80%)**	**1/5 (20%)**	**3/5 (60%)**	**2/5 (40%)**	**1/5 (20%)**	**1/5 (20%)**
**Overall: N = 42**	**2/42 (4.76%)**	**26/42 (61.90%)**	**19/42 (45.23%)**	**23/42 (54.76%)**	**20/42 (47.61%)**	**6/42 (14.28%)**	**9/42 (21.42%)**

## Discussion

At present, the main manifestations of spatial neglect are mainly detected through clinical observation and neuropsychological paper-and-pencil tests, that allow to explore its features (e.g., egocentric vs. allocentric, personal vs. extra-personal) in a standardized way, and to quantify its severity (Heilman et al., 2003; Lezak et al., 1995; Vallar & Bolognini, 2014). However, some pieces of evidence point out that, the longer the duration of the disease after stroke onset, the better the patients’ performances in neuropsychological tests, although difficulties are still found with ADLs (Bonato et al., 2010, 2012; Della Sala et al., 2018). This improvement of spatial neglect in stroke patients is due to both spontaneous recovery of neurological and neuropsychological deficits (Corbetta et al., 2005; Murphy & Corbett, 2009; Stefaniak et al., 2020; Stinear, 2017), and the effects of rehabilitation (Azouvi et al., 2017; Bowen et al., 2013; Kerkhoff & Schenk, 2012). Emerging literature suggests that IT-based devices could be an important tool for neuropsychological assessment. These technologies allow to record much more information with respect to paper-and-pencil tests, and to replicate and alter the surrounding world, permitting to evaluate the patients’ behavior in contexts resembling real life conditions (Ogourtsova et al., 2017; Pedroli et al., 2015; Tsirlin et al., 2009). Despite the potential of these technologies, several issues need to be taken into account when developing, disseminating and implementing CNADs (Bauer et al., 2012; see Kourtesis & MacPherson, 2021 for VR-related issues).

This scoping review aimed at presenting an overview of the studies in which IT-based devices were used as tools to assess manifestations of spatial neglect. The inclusion criteria were met by 42 articles, which were categorized into five groups, depending on their technological approaches: *computer* (13), *graphics tablet or tablet* (8), *virtual reality-based assessment* (16), and *other* (5).

### Computer (pc/laptop)

Computer tools have proved to be effective for the evaluation of manifestations of spatial neglect. These systems appear to be more sensitive in recording difficulties along the left-right spatial dimension, with respect to the gold standard and time-honoured paper-and-pencil tests. Many advantages of this kind of technology are acknowledged by the AACN and the NAN, as well as by this review. Relevant issues include the following: (i) testing large numbers of participants quickly (e.g., parallel administration); (ii) tests available at any time; (iii) enhanced accuracy and precision (e.g., reaction times, or exploration time of the two sides of space, Machner et al., 2018; Mizuno et al., 2016; Rabuffetti et al., 2012; Rossa et al., 2019; Villarreal et al., 2020, 2021); (iv) shorter administration times and reduced costs for test administration and scoring; (v) when appropriate, test versions for different languages; (vi) automatically exporting recorded data to databases (Chiba et al., 2010; Jee et al., 2015, for a digital version of the line bisection task); (vii) increased accessibility, for instance from remote; (viii) integration of algorithms for making decisions on issues such as the identification of an impairment or of a statistically reliable change in performance. These technologies can be easily integrated with other systems, thus providing a wider range of measures (e.g., Electroencephalography, Kocanaogullari et al., 2020). Finally, computerized tests adequately suit the purpose of being administered via telematics from remote: the currently ongoing SARS-CoV-2 world pandemic has emphasized this need.

On the other hand, the present qualitative evaluation of the studies points out several critical issues that must be addressed. With respect to sample size calculation, none of the studies applied power analyses. Ten studies included a control group of healthy participants, and four studies a control group of patients without spatial neglect. Only three out of 13 studies included both. This means that the ability of about 75% of the studies to distinguish different levels of performance among different groups of participants (healthy, affected or not affected by the deficit under investigation, as indicated by other tasks) was not completely assessed, making uncertain the possible clinical validity of most of the proposed IT-based tasks for distinguishing a defective from a preserved performance. As for psychometric properties, eight studies reported sensitivity or specificity measures; two studies report convergent or discriminant validity and only one ecological validity (only Machner et al., (2018) reported both). None of the studies reporting specificity or sensitivity, provided cut-offs for detecting performance impairments. Finally, with respect to user experience reporting, only Ulm et al. (2013) showed these data. The parameters of ecological validity and user experience reporting are most relevant, considering that ADL scales are most sensitive to detect spatial neglect (Azouvi, 2017) and the used IT-based devices are novel tools for both clinicians and patients.

### Graphic Tablet and Touch-Screen Tablet

The same list of *pros* discussed in the previous section readily applies to graphics and touch-screen tablets. The present qualitative evaluation raises several critical issues also for the studies included in this group. In the first place, sample size calculation based on power analysis was applied by only one study (Quinn et al., 2018). A control group of healthy participants was included in four studies and five studies included a control group of patients without spatial neglect; only two out of eight studies had both. This means, as for the computer group, that in about 75% of the studies, the possible clinical validity of most of the proposed IT-based tasks for distinguishing a defective from a preserved performance is uncertain. As for psychometric properties, sensitivity, or specificity measures and convergent or discriminant validity were more frequently reported than in the computer group: five studies reported sensitivity or specificity measures (62.5%) however, among them, only Chung et al. (2016) reported cut-offs for detecting impaired performances. Four studies (50%) reported convergent or discriminant validity. Ecological validity was underreported also in this group: only the article by Pallavicini et al. (2015) provided these measures. Finally, only two out of eight studies mentioned user experience reporting (Pallavicini et al., 2015; Quinn et al., 2018), showing high ratings of acceptance and usability of the *NeglectApp* and of the *StrokeVision app*.

### Virtual Reality

Among the 16 studies included in this group, non-immersive paradigms were used only in two studies (Buxbaum et al., 2012; Grattan & Woodbury, 2017). The *Virtual Reality Lateralized Attention Test (VRLAT)* consists indeed of a 2-dimensional screen presentation of a virtual, nonbranching path, in which participants interact with the environment using a joystick. The VRLAT detects manifestation of spatial neglect better than the traditional paper-and-pencil tasks; moreover, scores obtained at the VRLAT correlate with those obtained in the CBS and in the *Naturalistic Action Test* (Schwartz et al., 2002). However, Buxbaum et al. (2012) report that six out of 70 right brain-damaged patients (about 9%) did not complete the experimental procedure, due to difficulties in operating with the joystick; no other measures of user experience are provided. All the remaining 14 studies included in the VR group employed instead immersive paradigms: in particular, 10 studies integrated computers with head-mounted display, two used shutter glasses (Dvorkin et al., 2012; Fordell et al., 2011), and two body-tracking sensors (Mesa-Gresa et al., 2011; Sugarman et al., 2011). Regarding the methodological aspects, only Aravind and Lamongagne (2014) applied sample size calculation based on power analysis. Eight studies included a control group of healthy participants and eight a control group of patients without spatial neglect; six studies included both groups. As for psychometric properties, seven studies (43.75%) reported sensitivity or specificity measures, but only Buxbaum et al. (2012), also cut-offs for detecting performance impairments. Twelve studies reported instead convergent or discriminant validity measures (75%).

VR paradigms allow to replicate and alter the surrounding world to evaluate the patients’ behavior in contexts simulating real life (Ogourtsova et al., 2017; Pedroli et al., 2015; Tsirlin et al., 2009). However, only Buxbaum et al. (2012), who employed a non-immersive paradigm, and Kim et al. (2021), who used instead an immersive paradigm, provided ecological validity measures (12.5%). The paucity of measures regarding ecological validity is worth noticing, considering that one possible advantage of VR paradigms is to create scenarios that resemble real life situations, thus giving leeway to the evaluation of the patients’ behaviour in these contexts. In particular, the fact that only one out of 14 studies adopting immersive paradigms reported these measures is even more notable, considering that immersive VR technology allows to collect data through the employment of dynamic stimuli and interactions with a high degree of control within an ecologically valid environment (Kourtesis et al., 2020; Rizzo et al., 2004). Finally, with respect to user experience ratings, data, although encouraging, are few: as for non-immersive VR-paradigms, about 9% of the clinical sample did not complete Buxbaum et al.’s (2012) test, due to difficulties in operating with the joystick. As for the immersive VR-paradigms, Fordell et al. (2011) employed a three-item questionnaire: results show that, during the *VR-DiSTRO* battery administration, 41% of the clinical sample reported to feel focused, 22% to feel pleased during the administration of the task, and 25% to feel alert. Sugarman et al. (2011) made use of the *Short Feedback Questionnaire* (Rand et al., 2008): the only patient tested with the *VR react task* of the *SeeMe system* reported feelings of enjoy, control and success in the VR environment. In the study by Knobel et al. (2020), the administration of the *System Usability Scale* (Brooke, 1996) to the clinical sample revealed high ratings of acceptance and usability of their VR cancellation task. Conversely, Ogourtsova et al. (2018a, b) conducted focus groups and administered questionnaires directly involving the clinician: experts showed interest and availability to use VR paradigms in their clinical practice. Finally, only two studies investigated VRISE: no participant reported nausea or other adverse effects during or after the administration of the *VR-DiSTRO* battery of Fordell et al. (2011). Furthermore, cybersickness connected to the VR cancellation task of Knobel et al.‘s (2020) was rated as “very low” at the *Simulator Sickness Questionnaire* (Kennedy et al., 1993).

### Other

Studies included in this section employ an interactive table (Peru et al., 2017), a planar end-effector robot (*REAplan®robot*) (Montedoro et al., 2019), a tangible user interface system consisting in an integrated system of concrete objects that participants can manipulate (*E-TAN platform*) (Cerrato et al., 2020) and a driving simulator (Sotokawa et al., 2015; Spreij et al., 2020). None of the included study applied sample size calculation procedures. Four out of five studies included a control group of healthy participants, but only one (Spreij et al., 2020) also a control group of patients without neglect. Sensitivity or specificity measures were reported in three studies, convergent or discriminant validity measures in two. Only Spreij et al. (2020) reported measures of ecological validity. Finally, with respect to user experience, only Cerrato et al. (2020) provided the reports of some patients, who spontaneously described the task as agreeable and quick to perform, and as a welcome change after so many boring paper-and-pencil tests.

For sure, the simulated driving-based assessment offers an evaluation based on tasks like the situations people face in real life while driving; however, the two studies included in this section share the same limitations previously mentioned for VR paradigms. Furthermore, a requirement of driving simulators and VR devices is that both the experimenter and the participant have to be trained to be able to use them; this can be difficult for patients with cognitive impairment, such as memory deficits (Baddeley et al., 2003; Ferbinteanu, 2019). The same problem concerns all the innovative devices included in this section, that are realized with proprietary hardware and software components, since a specific training for each technological proposal is required.

Taken together, data emerging from the present qualitative synthesis of the studies included in this scoping review, although encouraging, are still few and leave than room for several critical issues. In the first place, from a methodological point of view, the recent literature points out the need to calculate the required sample size by using power analysis (Brysbaert, 2019; Kühberger et al., 2014). Accordingly, underpowered studies which may provide unclear results, and then the occurrence of publication biases, are to be avoided. However, despite the above mentioned methodolological critical issues, only two studies included in our review (about the 5%) used sample size calculation procedures. Furthermore, only 12 studies (less than 30%) included both a control group of healthy participants and patients without neglect: the comparison of the patients’ performances with those of a control group of participants, is indeed standard neuropsychological practice, to evaluate the performance of a patients’ sample with a specific diagnosis (in this review, spatial neglect), with respect to participants who do not show that specific impairment, namely: healthy control participants, patients without spatial neglect or both (Kan et al., 2019; Vallar, 2000). As for psychometric features, sensitivity, sensibility measures and convergent or divergent validity were reported in about 50% of the reviewed studies. Moreover, considering that in the subacute, and particularly in the chronic phase post-stroke, patients may still show difficulties in ADLs despite doing well in neuropsychological paper-and-pencil tests (Bonato et al., 2010, 2012; Della Sala et al., 2018), it is even more notable that ecological validity was addressed only in about 13% of the studies. Ecological validity is a subtype of external validity, that can be defined as the extent to which the measures of outcome correlate with, or predict, performance in ADLs (see also Holleman et al. 2020). Finally, despite user experience represents a further critical issue that should be addressed to benefit from the advantages of CNADs (Bauer et al., 2012; see Kourtesis and MacPherson, 2021 for VR-related issues), only about the 20% of the included studies reported user experience measures.

## Conclusion

The current findings make it difficult to establish golden standard tests or assessment procedures other than those (i.e., paper-and-pencil tests), that are extensively used in clinical settings for the evaluation of spatial neglect. Moreover, the process of developing IT-based tests in clinical neuropsychology should take into account the issues raised for the development, implementation and dissemination of CNADs and VR paradigms (Bauer et al., 2012; see Kourtesis & MacPherson, 2021 for VR-related issues). This is a laborious procedure, requiring the cooperation of a multidisciplinary team, including clinicians, researchers, informatics, interaction designers and engineers. While embracing technology is necessary, it is also essential to be aware of every aspect that leads to an efficient and effective assessment (see, for instance, Kourtesis & MacPherson, 2021). Nevertheless, it is our view that, through technical and user experience improvements, studies in brain-damaged patients and control participants, comparing paper-and-pencil and IT-based devices, and normative studies providing the cut-off scores, needed for the diagnostic process (e.g., Mancuso et al., 2015; Vallar et al., 1994), it will be possible to gather increasing evidence about the efficacy of at least some of the tests considered in this review. This, in turn, will provide additional support for changes and improvements in clinical practice, for diagnostic and follow up purposes. This review and the discussion of the available IT-based studies provides a database of information for the future developments of this line of research.

## Data Availability

data sharing not applicable to this article as no datasets were generated or analyzed during the current study.
